# Hubbard dimer physics and the magnetostructural transition in the correlated cluster material Nb_3_Cl_8_

**DOI:** 10.1038/s41699-026-00706-0

**Published:** 2026-05-20

**Authors:** Alberto Carta, Peter Mlkvik, Fabian Grahlow, Markus Ströbele, H.-Jürgen Meyer, Carl P. Romao, Nicola A. Spaldin, Claude Ederer

**Affiliations:** 1 PSI Center for Scientific Computing, Theory, and Data, Villigen, Switzerland; 2 Materials Theory, ETH Zürich, Zürich, Switzerland; 3 Section for Solid State and Theoretical Inorganic Chemistry, Institute of Inorganic Chemistry, Eberhard Karls Universität Tübingen, Tübingen, Germany; 4 Department of Materials, Faculty of Nuclear Sciences and Physical Engineering, Czech Technical University in Prague, Prague, Czech Republic

**Keywords:** Materials science, Physics

## Abstract

We present a combined computational and experimental study of Nb_3_Cl_8_, a correlated layered material containing Nb trimers, through the lens of competing intra- and intercluster interactions. Different proposed explanations for its magnetostructural transition, such as charge disproportionation, antiferromagnetic quenching, and interlayer singlet formation, are investigated in light of the various reported low-temperature structures. Our findings rule out the previously proposed charge-disproportionation, suggest an intricate interplay between Mott physics and the formation of interlayer singlets, and also hint at a possible explanation of the observed intratrimer scissoring distortion. We suggest that the physics of Nb_3_Cl_8_ should be understood in the context of weakly coupled Hubbard dimers.

**Figure:**
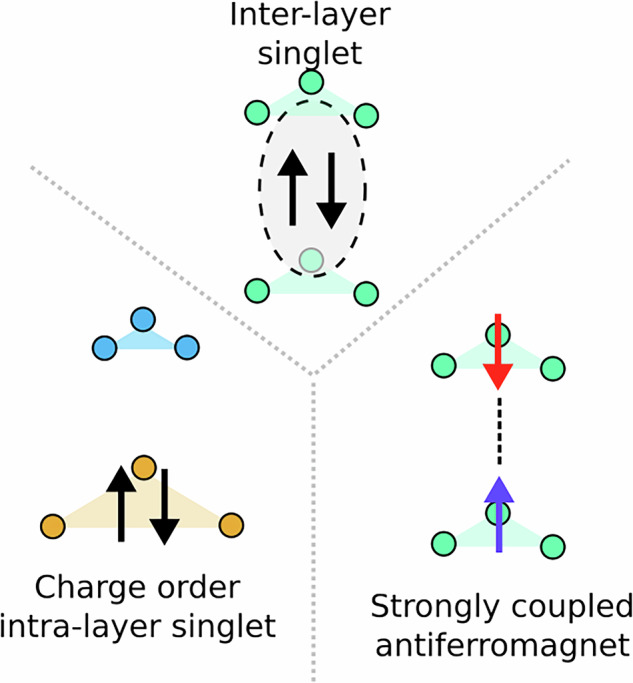

## Introduction

Cluster Mott insulators are strongly correlated materials for which the traditional definition of sites on which electrons interact^1^ is extended to include clusters of atoms^2–7^. The interplay of charge and spin degrees of freedom, combined with the complexity introduced by the crystal field of the cluster, makes these materials an exciting playground to explore and engineer the physics of strongly correlated systems.

Among these, Kagome cluster magnets^8^ have garnered considerable interest due to the interplay between magnetism and correlation effects. In this work, we focus on Nb_3_Cl_8_, part of the triniobium octahalide Nb_3_*X*_8_ (*X* = Cl, Br, I) family. These compounds have recently been recognized as possible spin-liquid candidates^9,10^ and as obstructed-atomic-insulator candidates^11^; they exhibit thickness-dependent conductivity^12^, display the anomalous valley Hall effect^13,14^, and have been showcased in Josephson diodes^15^ and infrared sensors^16^.

**Fig. 1:**
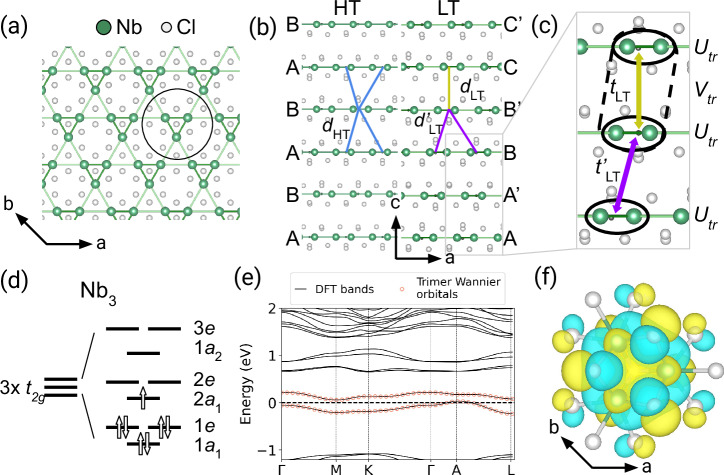
Bulk Nb_3_Cl_8_ as composed from monolayers. **a** Top view of the monolayer Nb_3_Cl_8_ with a trimer unit circled. **b** Side view of the bulk Nb_3_Cl_8_ in its HT and LT structures, showcasing the stacking and the change in distances between closest trimer centers across adjacent layers. **c** Close-up of the LT structure with the two relevant interlayer hoppings *t*_LT_ and ${t}_{\,{\rm{LT}}}^{{\prime} }$ indicated. *U*_tr_ and *V*_tr_ represent the intra- and inter-trimer interactions considered in our calculations. **d** Energy level diagram of the Nb_3_Cl_8_ trimer. **e** DFT band structure of LT bulk Nb_3_Cl_8_ with the Wannier bands superimposed. **f** Top view of the corresponding Wannier function for one of the trimers within an LT unit cell.

Nb_3_Cl_8_ is a layered 2D material consisting of Nb_3_Cl_13_ cluster units (circled in Fig. 1a) arranged in a breathing Kagome lattice^17–19^ with short intratrimer bond distances of *d*_Nb–Nb_ ≅ 2.81 Å (see Supplementary Table 1). The nominal electronic filling of each trimer is 7 electrons, and due to the octahedral coordination of the Nb ions in the cluster crystal field, the energy levels are split, leaving a lone electron in the 2*a*_1_ orbital (as shown in Fig. 1d). In both the monolayer and bulk forms, these *a*_1_ orbitals form narrow bands close to the Fermi level which are well separated from the rest of the electronic bands as shown in Fig. 1e. Wannier functions corresponding to these *a*_1_ states can be constructed as triangular molecular orbitals of mixed Nb–halogen character which are extended over the whole trimer^20^ as seen in Fig. 1f.

While the free-standing monolayer Nb_3_Cl_8_ has been characterized as a 2D Mott insulator both experimentally^21–23^ and theoretically^9,20,21,24,25^, the nature of the bulk material remains less explored. At high temperature (HT), bulk Nb_3_Cl_8_ is found in the *α*-phase with space group $P\bar{3}m1$, with two consecutive layers stacked in an AB-staggered pattern^12,15,19,26–28^. The interlayer distance is ~6.8 Å while the intertrimer distance (distance between centers of adjacent Nb_3_ trimers in the stacking direction) is *d*_HT_ ≅ 7.8 Å^27^ (Fig. 1b, left).

As the temperature is lowered below *T*_c_ ≅ 100 K, the material remains insulating but undergoes a magnetostructural transition to the low-temperature (LT) *β*-phase, which exhibits a significant change in the stacking pattern to an -AA’-BB’-CC’- arrangement^27,29,30^. This results in a “dimerization” of nearest-neighbor trimers along the stacking direction (Fig. 1b, right)^27,30^. The newly formed pairs’ intertrimer distance decreases significantly to *d*_LT_ ≅ 6.1 Å (see Supplementary Table 1), and the calculated intertrimer hopping integral increases from 0.01 to *t*_LT_ ≅ 0.13 eV (see Supplementary Table 2).

The space group of the LT *β*-Nb_3_Cl_8_ is still a matter of debate, with several different candidate structures proposed. Kim et al.^31^, for instance, report $R\bar{3}m$ symmetry corresponding to the ideal version of this stacking, which has also been observed in other members of the extended Nb_3_X_8_ family: Nb_3_Cl_4_Br_4_^29^, Nb_3_Br_8_^32^, and Nb_3_I_8_^31^. In the literature, lower symmetry structures such as *R*3^27,33^ and *C*2/*m*^30^ have also been proposed.

The different LT phases reported in the literature could plausibly be ascribed to differing experimental conditions used to synthesize and characterize Nb_3_Cl_8_ and related phases. The *R*3 and *C*2/*m* phases have been observed using single crystal diffraction techniques^27,30^, which are generally highly sensitive to crystallographic symmetry, whereas $R\bar{3}m$ was assigned to the LT phase using a combination of experimental and theoretical vibrational spectroscopy^31^, which is a less reliable method of determining the space group. Nb_3_Cl_4_Br_4_ was also observed to adopt an $R\bar{3}m$ structure at low temperature using single crystal diffraction^29^.

In all cases, the structural transition is accompanied by a significant drop in the magnetic susceptibility^27,29,30,33^, indicating a strong quenching of the local trimer moments. Several interpretations have been proposed to explain the observed drop in magnetic susceptibility. Haraguchi et al.^27,33^ report a “breathing” effect in the Nb_3_ trimers, with alternating layers of shorter and longer Nb–Nb bond lengths in the *R*3 structure from single-crystal X-ray diffraction (XRD). The authors propose charge disproportionation, $2{[{{\rm{Nb}}}_{3}]}^{7}\to {[{{\rm{Nb}}}_{3}]}^{8}+{[{{\rm{Nb}}}_{3}]}^{6}$, leading to the formation of a singlet on the larger Nb trimers and causing a drop in the magnetic susceptibility^27^.

When comparing Nb_3_Cl_8_ to other materials that exhibit singlet formation or charge disproportionation and subsequent structural distortions, several issues arise with the charge disproportionation model. Haraguchi et al.^27,33^ report bond lengths of *d*_Nb1–Nb1_ = 2.801(3) and *d*_Nb2–Nb2_ = 2.821(2) Å for the two inequivalent trimers, resulting in a notably small Nb–Nb bond-length difference of just 0.02 Å. We can compare these small distortions with other materials where metal-metal bonding is important. Vanadium dioxide (VO_2_), for instance, is a material undergoing a similar paramagnetic-to-nonmagnetic transition associated with the formation of a V–V singlet state^34,35^. VO_2_ features a magnetostructural transition (coupled to a metal–insulator transition), contracting the original V–V bond by almost 10% or ~0.3 Å^36^, roughly 15 times that observed in ref. ^27^ for Nb_3_Cl_8_.

Moreover, using neutron diffraction, Sheckelton et al.^30^ observe a different LT Nb_3_Cl_8_ structure which still exhibits the same magnetic-to-nonmagnetic transition, but has the *C*2/*m* space group. This phase is characterized by an isosceles triangle geometry within the Nb_3_ trimers, leading to bond distances of *d*_Nb1’–Nb2’_ = 2.86(2) and *d*_Nb2’–Nb2’_ = 2.72(1) Å^30^. However, the authors ascribe the change of intratrimer distances to secondary effects and speculate that it is the stacking of the *β* phase that drives the formation of singlets between different layers, causing the observed drop in susceptibility^30^.

Computationally, due to the small bandwidth and thus the potential importance of local electron–electron interactions, Nb_3_Cl_8_ has largely been studied using a combination of density-functional theory (DFT) and dynamical mean-field theory (DMFT)^20,21,24,37^ or similar beyond-DFT methods^9,38^. The bulk HT *α*-Nb_3_Cl_8_ has been labeled as a cluster-Mott insulator with many similarities to the 2D precursor material^9,20,21,24^, consistent with the isolated local moments of the experimentally observed HT Curie–Weiss susceptibility. For the LT *β*-Nb_3_Cl_8_ phase, most works agree on the important role of the increase in interlayer coupling [*t*_LT_ versus ${t}_{\,{\rm{LT}}}^{{\prime} }$ in Fig. 1c, see also Supplementary Table 2], even though the specific proposals for the relevant physics vary.

By evaluating the role of an intratrimer Coulomb interaction correction *U*_tr_, studies in bilayer LT *β*-Nb_3_Cl_8_^21,24^ conclude that it is the competition between the interlayer hopping, *t*_LT_, and intratrimer Coulomb interaction that determines whether the system is an interlayer singlet insulator or a Mott insulator, with strong interlayer antiferromagnetic fluctuations. This conclusion has been bolstered by the studies on bulk systems from Grytsiuk, Aretz et al.^20,37^. The same authors also report a change from a Mott-insulating hypothetical Nb_3_F_8_ to a weakly correlated Nb_3_I_8_, due to the decreasing onsite Coulomb interaction *U*_tr_ and the increasing interlayer hopping. Although the authors perform calculations of the experimentally observed *R*3 and the *C*2/*m* structures^20^, the origin and stability of the *β* Nb_3_Cl_8_ low-symmetry phases with respect to the $R\bar{3}m$ phase remain unclear.

In our work, we employ both DFT + *U* + *V*^39^ and DFT+DMFT^40^ to assess and analyze the different mechanisms proposed for the observed magnetostructural transition using a basis of trimer-centered Wannier orbitals. The larger spatial extent of such molecular trimer orbitals in comparison to more localized atomic-like orbitals leads to a non-negligible intertrimer Coulomb repulsion *V*_tr_^37^. We hence consider both types of interactions, *U*_tr_ and *V*_tr_, and, in particular, focus on analyzing their influence on the physics of the bulk structure.

Our investigation of the different gap opening mechanisms due to intra- and intercluster interactions in the LT *β* phase shows a clear disagreement with the charge disproportionation picture^27,33^. Instead, we obtain a continuous crossover between a Mott-insulating regime with strong antiferromagnetic intertrimer correlations and a band-insulator corresponding to the formation of intertrimer singlets^21,24,30,37^. We conclude that the physics of LT Nb_3_Cl_8_ is well described within a picture of weakly coupled Hubbard dimers. Additionally, we also suggest a possible origin of the scissoring distortion reported in Sheckelton et al.^30^, resulting from atomic-level interactions within the trimer. We corroborate our theoretical results with an experimental investigation of the structure and properties of Nb_3_Cl_8_ and Nb_3_Cl_4_Br_4_, and suggest that synthesis conditions influence the LT phases of Nb_3_Br_4−*x*_Cl_4+*x*_ materials.

## Results

### Stability of the *R*3 charge-ordered phase

We begin by investigating the stability of the proposed trimer breathing mode coupling to charge order as described by Haraguchi et al.^27,33^.

To do so, we construct molecular trimer Wannier functions from the 2*a*_1_ bands that lie close to the Fermi level (Fig. 1e) following ref. ^20^. We then perform a series of DFT + *U* + *V* calculations using these trimer orbitals as the basis, treating both the Coulomb interactions on one trimer (*U*_tr_) and between adjacent trimers (*V*_tr_). This is made possible by a recently developed extension of the QUANTUM ESPRESSO code^41^.

**Fig. 2:**
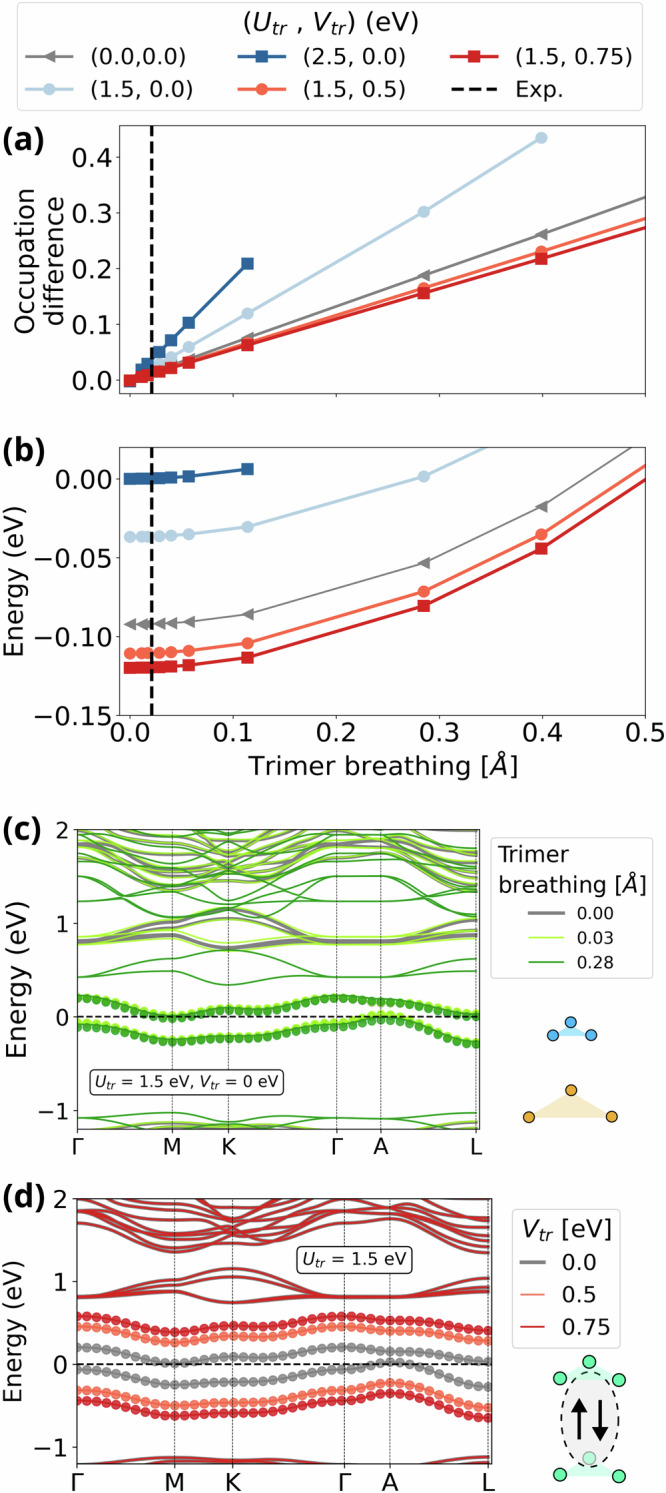
Behaviour of bulk Nb_3_Cl_8_ under the effects of *U* and *V*. **a** Occupation difference between trimers and **b** total energy per unit cell as a function of the breathing mode for different values of *U*_tr_ and *V*_tr_. The vertical dashed line indicates the experimentally reported amplitude of the breathing mode from ref. ^27^. Band structure plots for **c** three breathing mode values at (*U*_tr_, *V*_tr_) = (1.5, 0) eV and for **d** zero breathing mode ($R\bar{3}m$ structure) at three *V*_tr_ values for *U*_tr_ = 1.5 eV. The trimer bands are highlighted with full circles.

The proposed breathing mode (see inset graphic in Fig. 2c) breaks the symmetry between the two trimers in the unit cell, leading to a difference in their onsite single-particle energies. Specifically, the energy of the long-edge trimer is lowered, while that of the short-edge trimer is raised.

In this section, we focus on spin unpolarized calculations, where the onsite occupation for spin up equals the onsite occupation for spin down, ${n}_{ii}^{\uparrow }={n}_{ii}^{\downarrow }:={n}_{ii}$. The spin-polarized case will be treated in the next section. In this case, the Hubbard contribution to the Kohn–Sham potential due to the onsite interaction parameter reads:

1 $$\Delta {v}_{ii}^{\sigma }={U}_{{\rm{tr}}}\left(\frac{1}{2}-{n}_{ii}\right).$$

In the high-symmetry $R\bar{3}m$ structure, the two trimers are equivalent, leading to an onsite occupation of exactly *n*_*ii*_ = 0.5 for each one. Consequently, the potential correction from Eq. (1) is zero. A structural distortion, such as the trimer breathing mode that lowers the symmetry to *R*3, breaks this equivalence. This splitting of the trimer orbital energy levels creates a small initial occupation difference, Δ*n*. The Hubbard *U* term then acts to amplify this imbalance: for the more occupied trimer (*n*_*ii*_ > 0.5), $\Delta {v}_{ii}^{\sigma }$ is negative, favoring further filling, while for the less occupied one (*n*_*ii*_ < 0.5), $\Delta {v}_{ii}^{\sigma }$ is positive, promoting emptying. Following the charge disproportionation model proposed by Haraguchi et al.^27^, a sufficiently strong Hubbard *U* can be expected to drive this system towards a larger occupation difference, potentially approaching the limit of two electrons, thereby forming a singlet state on one trimer while completely emptying the other. However, our calculations reveal that this amplification effect is rather weak, as also manifested by the small onsite energy difference due to the trimer breathing distortion (see Supplementary Fig. 1).

Our results are presented in Fig. 2a, b, where we plot the occupation difference and the energy as a function of the trimer breathing mode amplitude, which is defined as the edge difference between long-edge and short-edge trimers, for values around the calculated constrained random-phase approximation (cRPA) values [(*U*_tr_, *V*_tr_) = (1.46, 0.38) eV, see Supplementary Table 2]. We explicitly vary the trimerization distortion mode and calculate the energy separately at every step. We remark that the absolute value of the energy difference between calculations with different *U*_tr_ and *V*_tr_ is not physically meaningful, and it is only shown in Fig. 2b for visual clarity.

We find that for (*U*_tr_, *V*_tr_) = (1.5, 0.0) eV, the reported *R*3 structure^27,33^ corresponds to an occupation difference of only 0.03 electrons (see vertical dashed line in Fig. 2a). We observe that even at *U*_tr_ = 2.5 eV (60% higher than the computed cRPA value) and at a breathing mode amplitude of 0.4 Å (20 times the experimental value), the occupation difference remains smaller than 0.4 electrons. Furthermore, we observe that the energy minimum consistently occurs at zero-breathing mode, which corresponds to the higher-symmetry $R\bar{3}m$ structure (Fig. 2b).

By examining the band structure at different breathing mode amplitudes (Fig. 2c), we observe minimal changes in the bands near the Fermi level as the breathing-mode amplitude increases, and the material remains metallic. This finding is not consistent with the experimentally observed insulating behavior.

To investigate this point further, we perform calculations where, in addition to the +*U* correction, we include an intersite term *V*_tr_. The +*V* correction essentially modifies the effective hopping between neighboring sites by adding an intersite potential^39^:

2 $$\Delta {v}_{ij}^{\sigma }=-{V}_{{\rm{tr}}}{n}_{ji}^{\sigma }\,.$$

Here, ${n}_{ji}^{\sigma }$ is the off-diagonal intertrimer component of the density matrix, which is a measure of the intersite hybridization.

Upon including *V*_tr_ (light red and red lines in Fig. 2a, b), we observe a further suppression of the occupation difference between trimers. This is accompanied by an opening of a band gap (Fig. 2d), the nature of which will be discussed in more detail in the next section.

Consequently, based on our DFT + *U* + *V* calculations, the charge-ordered phase, accompanied by a trimer breathing mode as proposed by Haraguchi et al.^27^, appears to be energetically unfavorable. We therefore rule out the charge disproportionation mechanism as an explanation for the observed magnetic-nonmagnetic transition in Nb_3_Cl_8_.

### Origin of the quenched magnetism in the $R\bar{3}m$ phase

We now consider different possible explanations of the low susceptibility observed in *β*-Nb_3_Cl_8_. In this section, we study separately the effects of the interaction terms *U*_tr_ and *V*_tr_, allowing for spin-polarization in the system.

As previously mentioned, with no spin-polarization, one would expect the +*U* correction to promote charge polarization in one of the trimer orbitals. In spin-polarized calculations, instead, the +*U* correction can favor the formation of an onsite magnetic moment on the Nb_3_ molecular orbital. This is because the potential shift $\Delta {v}_{ii}^{\sigma }={U}_{{\rm{tr}}}\left(\frac{1}{2}-{n}_{ii}^{\sigma }\right)$ for site *i* and spin *σ*, promotes either fully occupied or completely empty spin channels on each orbital. So, at strong *U*, one would expect one trimer orbital one spin channel *σ* to be completely occupied ${n}_{ii}^{\sigma } \sim 1$ and the opposite spin channel $\bar{\sigma }$ to be empty ${n}_{ii}^{\bar{\sigma }} \sim 0$, which corresponds to the formation of a local magnetic moment on the trimer.

**Fig. 3:**
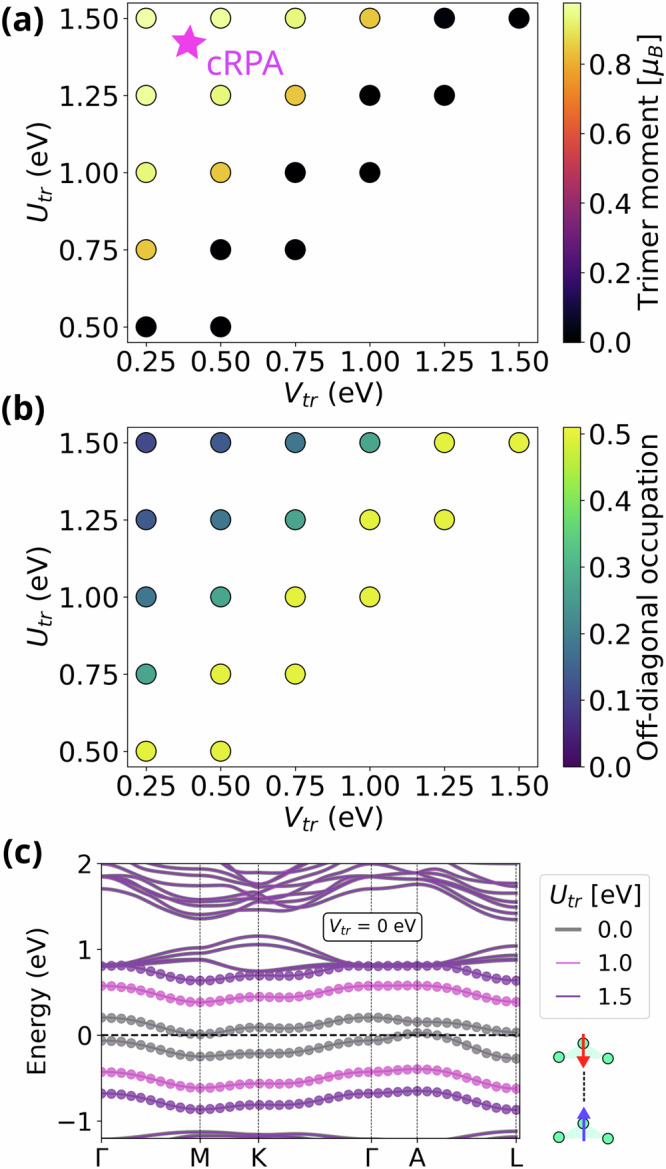
Behaviour of the magnetic moments of Nb_3_Cl_8_ under the effects of *U* and *V*. **a** Absolute value of the trimer moment and **b** intertrimer occupation as a function of *U*_tr_ and *V*_tr_. Star indicates the cRPA values. **c** Band structure within spin-polarized DFT + *U* + *V* with antiferromagnetically aligned trimer magnetic moments for three *U*_tr_ values for *V*_tr_ = 0. Bands corresponding to the trimer orbitals are highlighted with circles. All calculations are performed for the $R\bar{3}m$ structure (no trimer breathing mode).

In Fig. 3a, b, we show the average onsite trimer moment $| {n}_{ii}^{\uparrow }-{n}_{ii}^{\downarrow }|$ and the magnitude of the off-diagonal occupation matrix element ${n}_{ij}^{\sigma }$, respectively, as a function of *U*_tr_ and *V*_tr_. In Fig. 3c, we show the band structure for fixed *V*_tr_ and changing *U*_tr_.

Two competing tendencies emerge: a strong onsite Coulomb interaction *U*_tr_ favors the development of antiferromagnetically aligned local magnetic moments on the Nb_3_ sites (Fig. 3a), suppressing any intersite bonding (${n}_{ij}^{\sigma }\to 0$ in Fig. 3b).

This local moment formation leads to a band gap opening (Fig. 3c) and the strong antiferromagnetic correlations between adjacent trimers could be responsible for the suppression of the magnetic susceptibility^20^. We underline that we are not arguing for the presence of long-range antiferromagnetic order in the material. Rather, we argue that in the strong *U*_tr_ regime, nearest-neighboring trimer orbitals could show strong antiferromagnetic correlations, as schematically depicted in the inset in Fig. 3c. Conversely, a dominant intersite Coulomb interaction *V*_tr_ promotes the formation of bonding-antibonding states between trimers (${n}_{ij}^{\sigma }=0.5$) (Fig. 3b) and also leads to the formation of a band gap (as was previously shown in Fig. 2d), which in this case is related to the large bonding–antibonding splitting. Since two electrons per dimer would then occupy the lower-lying bonding state, forming a nonmagnetic singlet (inset in Fig. 2d), this scenario would also be compatible with a drop in magnetic susceptibility.

Our cRPA calculations for the trimer molecular orbitals, in good agreement with the findings of Grytsiuk, Aretz et al.^20,37^, suggest that Nb_3_Cl_8_ lies closer to the strong *U*_tr_ regime.

To summarize, our calculations indicate that a change in stacking from $P\bar{3}m1$ to $R\bar{3}m$ is sufficient to explain the low magnetic susceptibility observed in *β*-Nb_3_Cl_8_, without the need to introduce any further symmetry-lowering distortions, in agreement with previous works^20,37^.

### Dynamical effects in the $R\bar{3}m$ phase

So far, our treatment of the physics in Nb_3_Cl_8_ has been based on a static mean-field treatment of interaction effects, which highlights the crucial role of both onsite and intersite interactions, particularly within dimerized units of Nb_3_ molecular orbitals. To move beyond this static mean-field approximation and capture dynamical effects, we now employ DFT+ cluster DMFT, which has been shown to be a particularly viable approach for studying quantum singlet-like states^37,42^.

We construct a DMFT impurity problem for the $R\bar{3}m$ structure using a cluster of two neighboring molecular trimer orbitals, as first introduced in Aretz et al.^37^, and explore the behavior of the system under a variety of interaction parameters, in particular exploring the nature of the insulating state.

This approach is well suited for systems like Nb_3_Cl_8_ due to the strong onsite and intersite interactions *U*_tr_ and *V*_tr_, a significant intertrimer hopping along the stacking direction (*t*_LT_ = 0.13 eV, see Supplementary Table 2, and comparatively weaker hybridization between the dimerized cluster and the rest of the Nb_3_ molecular orbitals in plane (${t}_{{\rm{LT}}}^{{\rm{in-plane}}}=0.02$ eV, see Supplementary Table 1). These considerations allow us to draw significant analogies between the physics in the LT phase of the Nb_3_*X*_8_ family and the so-called *Hubbard dimer*, a model system consisting of two interacting sites with one orbital each.

For two electrons in the Hubbard dimer model (i.e., at half filling), the gap, *E*_g_, in the excitation spectrum between the highest occupied and lowest unoccupied states of the Hubbard dimer, is dependent on the three parameters that define the model, the intersite hopping *t*, the onsite *U* and the intersite *V* interaction parameters^43^:

3 $${E}_{{\rm{g}}}=-2t+V+\sqrt{{(U-V)}^{2}+16{t}^{2}}.$$

In general the (unnormalized) ground state of such a model system $\left|{\psi }_{0}^{\,{\rm{dimer}}}\right\rangle$ can be written as

4 $$\begin{array}{cl}\left|{\psi }_{0}^{\,\mathrm{dimer}}\right\rangle & =A\left(\left|\uparrow ,\,\downarrow \right\rangle -\left|\downarrow ,\,\uparrow \right\rangle \right)\\ & +\left(\left|\uparrow \downarrow ,\,0\right\rangle +\left|0,\,\uparrow \downarrow \right\rangle \right),\end{array}$$

where $A=4t/[\sqrt{{(U-V)}^{2}+16{t}^{2}}-(U-V)]$. Here, $\left|\uparrow ,\,\downarrow \right\rangle$ corresponds to a state with an up-spin electron on the first and a down spin electron on the second site, whereas $\left|\uparrow \downarrow ,\,0\right\rangle$ corresponds to double occupation of the first site, and analogous for the other states in Eq. (4).

For *U* = *V*, *A* = 1, the ground state can be described by a single Slater determinant expressed in the bonding–antibonding basis and corresponds to the bonded state of two trimer sites. The opposite limit, *U* ≫ *V*, *A* → *∞*, corresponds to the suppression of double occupancies and the maximally correlated state, the Heitler–London limit.

**Fig. 4:**
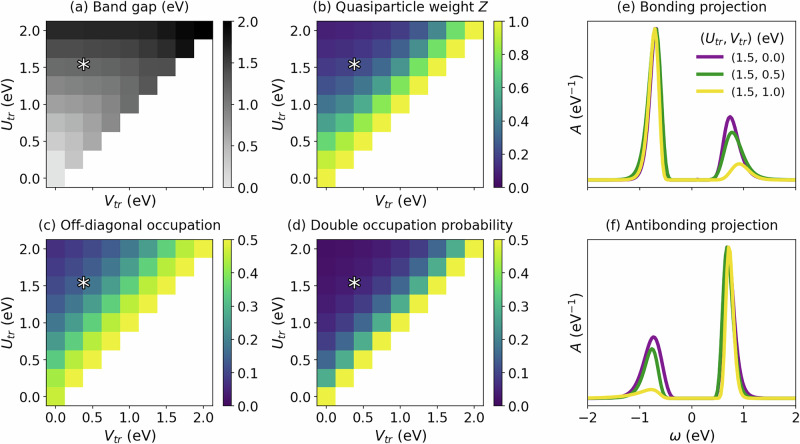
Observables of the $R\bar{3}m$ Nb_3_Cl_8_ cluster from DFT + DMFT as a function of *U*_tr_ and *V*_tr_. The cRPA values for Nb_3_Cl_8_ are shown with a star. **a** Band gap. **b** Quasiparticle weight, *Z*. **c** Off-diagonal occupation. **d** Double occupation probability. **e** Spectral function projected onto the bonding and **f** antibonding combination of the individual trimer orbitals across the (*U*_tr_, *V*_tr_) spectrum.

In Fig. 4a–d, we depict the dependence of various properties of *β*-Nb_3_Cl_8_ on both *U*_tr_ and *V*_tr_. We show the band gap (Fig. 4a) obtained from the spectral function, and the quasiparticle weight (Fig. 4b) defined from the frequency dependence of the self-energy. In addition, we also show the off-diagonal occupation matrix element (Fig. 4c) and the probability of double occupancy on one of the Nb_3_ molecular orbitals (Fig. 4d). The latter is obtained from analyzing the probability of the various multiplet states occurring in the two-site cluster, based on the statistics from the employed quantum Monte Carlo solver.

We obtain an insulating solution for any value of the tested *U*_tr_ and *V*_tr_ parameters, except in the vicinity of *U*_tr_ = *V*_tr_ = 0 (Fig. 4a), with the size of the band gap depending on both *U*_tr_ and *V*_tr_. At high *U*_tr_ and small *V*_tr_, the insulator exhibits hallmark features of a Mott state, such as a vanishing quasiparticle weight (Fig. 4b). By increasing *V*_tr_/*U*_tr_, the quasiparticle weight rises, reaching *Z* = 1 at *U*_tr_ = *V*_tr_, where the system is a band insulator. This is accompanied by a gradual increase in the off-diagonal occupation from *V*_tr_ = 0 to the maximum 0.5 at *U*_tr_ = *V*_tr_, with the double-occupation probability changing in a similar way (Fig. 4c, d). At small *V*_tr_/*U*_tr_, our results show a marked suppression of double occupancies, highlighting the localization of a single electron per Nb_3_ site. The multiplet analysis can also clarify the relative spin orientation of the Nb_3_ sites. Even at small *V*_tr_/*U*_tr_, configurations with spins aligning in opposite directions between trimers have a high probability, while configurations where spins point in the same direction on both trimers are suppressed.

The analogies to the Hubbard dimer are thereby particularly evident. We can identify the band insulator observed on the diagonal in Fig. 4a–d with the *A* = 1 bonding regime and the Mott insulator at low *V*_tr_/*U*_tr_ with the correlated high *A* regime. Importantly, our results indicate a continuous crossover between the two different insulating states of the LT phase, which is reflected in all observables examined.

Finally, we further exemplify this evolution by showing the spectral function at a fixed value of *U*_tr_ and different values of *V*_tr_, projected on suitably constructed bonding and antibonding dimer orbitals (Fig. 4e, f). We observe that the two peaks change their character with increasing *V*_tr_. The lower peak becomes predominantly bonding, while the upper peak becomes more antibonding as *V*_tr_ increases. Due to inherent difficulties in the analytic continuation and the large size of the gap, the spectral function shows smooth peaks across the spectrum, and we are not able to resolve finer spectral details such as the splitting of the Hubbard peaks that was observed, e.g., in the work of Aretz et al.^37^.

These results provide evidence that the physics of the $R\bar{3}m$ phase of Nb_3_Cl_8_ and the Nb_3_*X*_8_ family in general can be described by dimers of Nb_3_ molecular units, which are weakly coupled to each other. These findings support the results from the previous sections and indicate the presence of strong local antiferromagnetic correlations, which could be ultimately responsible for the suppression of the magnetic susceptibility in *β*-Nb_3_Cl_8_, as has been suggested previously^37^. The additional analysis of the *U* and *V* dependence sheds light on the evolution of this state under stronger electronic correlation effects. Moreover, we explicitly demonstrate a continuous crossover between the Mott and singlet (band) insulating states, in line with recent theoretical proposals^24,44,45^. The cRPA calculations indicate that Nb_3_Cl_8_ itself lies closer to the Mott limit, while the rest of the family lies closer to the singlet (band) insulating limit [see Supplementary Table 3 and Supplementary Fig. 3 for the rest of the family].

### Further symmetry lowering in the *β*-Nb_3_Cl_8_ phase

**Fig. 5:**
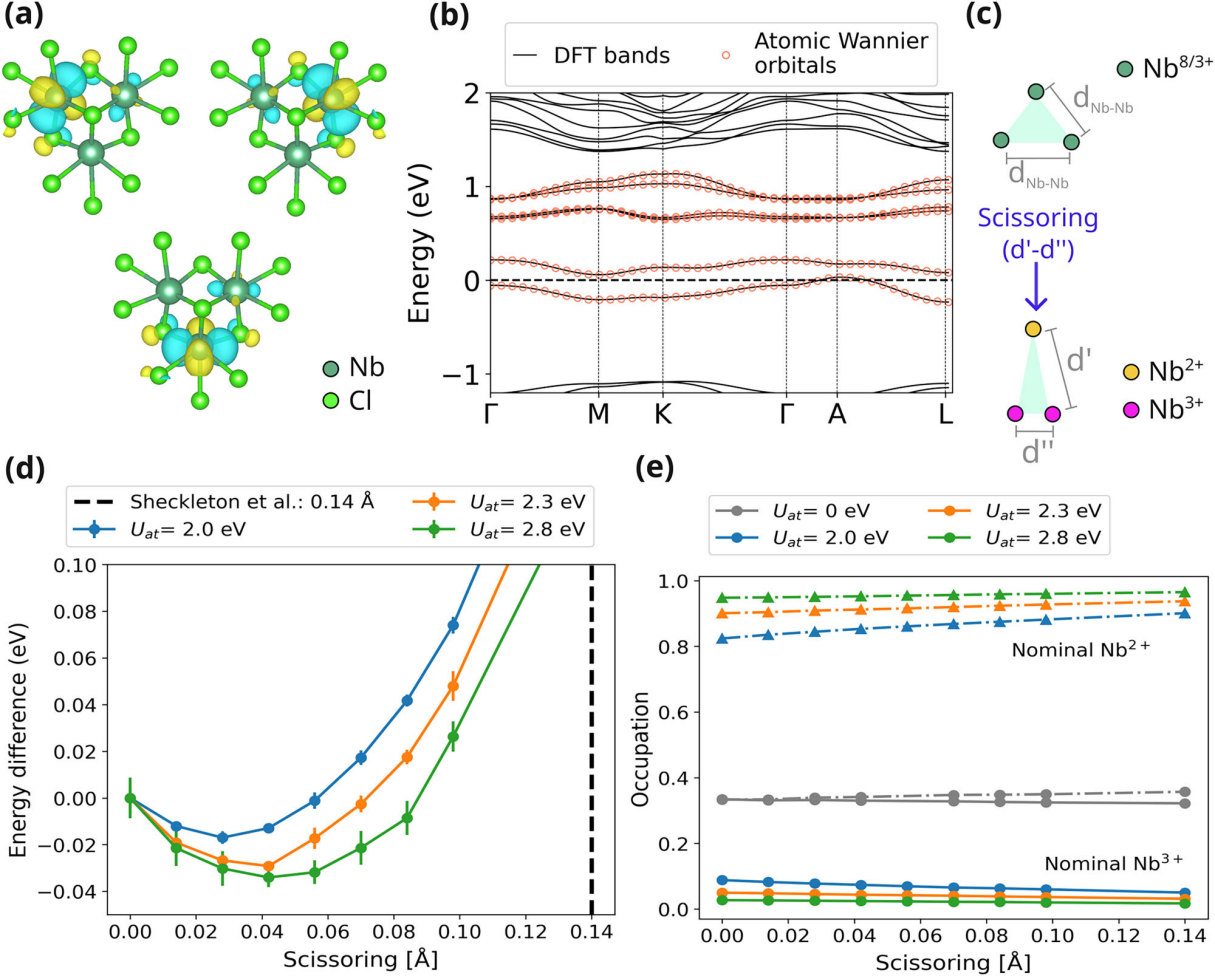
Behaviour of β-Nb_3_Cl_8_ under symmetry lowering. **a** Top view of the three atomic Wannier functions per trimer. **b** DFT band structure with the bands recalculated from the atomic Wannier model. **c** Schematic of the scissoring distortion within one trimer, connecting the $R\bar{3}m$ structure to the *C*2/*m*. **d** Energy difference per unit cell as a function of the scissoring amplitude. The black dashed line corresponds to the scissoring amplitude reported in ref. ^30^. **e** Occupation of the inequivalent Nb atoms inside one trimer. The solid lines correspond to the symmetry-equivalent nominal Nb^3+^ sites, while the dash-dotted line corresponds to the nominal Nb^2+^ site.

We will now consider the case of a system very close to the Mott regime, with relatively little intertrimer hybridization as a starting point to understand further symmetry-lowering effects, analyzing the physics within the Nb_3_ unit. By incorporating all six bands between 1.2 and −0.5 eV (as shown in Fig. 5b, corresponding to the 2*a*_1_ and 2*e* levels in Fig. 1d) in the wannierization, we construct a basis set consisting of one *d* atomic orbital per Nb atom, yielding a total of three *d* orbitals per Nb_3_ trimer (as shown in Fig. 5a) and six *d* orbitals per unit cell^46^.

Using cRPA, we calculated the relevant, now atomic, interaction parameters (see Supplementary Table 3): the onsite interaction on a single Nb atom, *U*_at_ = 2.3 eV, the intratrimer interaction between two different Nb atoms inside the same trimer, ${V}_{\,{\rm{at}}}^{{\rm{intra}}}=1.36$ eV, and the intertrimer interaction between neighboring trimers, ${V}_{\,{\rm{at}}}^{{\rm{inter}}}=0.39$ eV.

Leveraging our previous finding that the intertrimer occupation is small in the Mott regime, leading to electron localization on the trimer, we neglect the interaction across trimers (${V}_{\,{\rm{at}}}^{{\rm{inter}}}$) for simplicity. We then perform DFT + DMFT calculations considering two separate impurity problems, with each impurity containing the three Nb *d* orbitals, including *U*_at_ and ${V}_{\,{\rm{at}}}^{{\rm{intra}}}$ in the interaction Hamiltonian (see Eq. (8) for further details).

While DFT calculations distribute the single electron per each trimer equally among the Nb atoms, leading to an oxidation state of Nb^8/3+^, in our charge self-consistent DFT + DMFT calculations, we observe that for realistic values of $({U}_{{\rm{at}}},{V}_{{\rm{at}}}^{{\rm{intra}}})$ most of the charge concentrates on one of the Nb atoms (see Fig. 5e for 0.0 Å scissoring). This charge ordering within the trimer changes the nominal oxidation states of the three Nb atoms and renders them inequivalent: for every Nb_3_ trimer, we obtain one nominal Nb^2+^ site and two empty Nb^3+^ sites.

Our results also indicate that the structure accommodates this change in oxidation state within each trimer by elongating the bond length $d^{\prime}$ between the nominal Nb^3+^ and Nb^2+^ atoms and contracting the bond length *d″* between the two Nb^3+^ atoms (see Fig. 5c). The difference, $d^{\prime} -d^{\prime\prime}$, is termed *scissoring*. This scissoring distortion lowers the symmetry from $R\bar{3}m$ to *C*2/*m*, precisely matching both our own (see next section) and previous experimental work by Sheckelton et al.^30^.

This can be seen from Fig. 5d, e, where we show the energy per unit cell and the occupation of the nominal Nb^2+^ and Nb^3+^ calculated at every step of the scissoring mode. We consider various values of *U*_at_, while maintaining a constant ratio of ${V}_{\,{\rm{at}}}^{{\rm{intra}}}/{U}_{{\rm{at}}}=0.24$ (corresponding to our cRPA results). However, it is worth noting that our results exhibit weak dependence on the value of ${V}_{\,{\rm{at}}}^{{\rm{intra}}}$, and similar outcomes can be obtained by setting ${V}_{\,{\rm{at}}}^{{\rm{intra}}}=0$.

For *U*_at_ = 0 eV, the scissoring changes the occupation of the Nb atoms only slightly (Fig. 5e, gray line), and the system always relaxes back to $R\bar{3}m$ symmetry. For all finite values of *U*_at_ considered, the system shows strong electron charge concentration on the nominal Nb^2+^ atom (Fig. 5e, colored lines) and an energy minimum at a finite scissoring distortion (Fig. 5d). As *U*_at_ increases, the energy minimum deepens and shifts towards a larger scissoring amplitude, while the occupation of the Nb^2+^ atom becomes closer to 1. The scissoring distortion hence increases the onsite energy difference between the nominally Nb^2+^ and Nb^3+^ atomic sites (see Supplementary Fig. 2).

Compared to the results of Sheckelton et al.^30^, our calculated equilibrium scissoring amplitude is markedly smaller. For *U*_at_ = 2.3 eV (orange solid line in Fig. 5d), we find ~0.04 versus 0.14 Å reported in ref. ^30^ (indicated by the vertical black line in Fig. 5d).

As a remark, we also note that comparable results can be obtained through simpler DFT + *U* calculations, utilizing standard orthonormalized atomic orbitals as implemented in QUANTUM ESPRESSO^47^. If one starts an atomic relaxation with a small scissoring distortion and initializes the entire magnetic moment on a single Nb atom per Nb_3_ trimer (and ensures that the two magnetic Nb atoms in a unit cell containing two trimers are initialized with opposite magnetization), then for realistic values of the Hubbard *U* parameter, the system relaxes to *C*2/*m* symmetry. This *C*2/*m* phase is lower in energy than the antiferromagnetic $R\bar{3}m$ phase, where the magnetization is uniformly spread over the trimer, and it exhibits a scissoring mode amplitude similar to that found in our DFT + DMFT calculations, with most of the trimer’s magnetization concentrated on the nominal Nb^2+^ atom. We note that in the DFT + *U* calculations, an artificial antiferromagnetic order has to be imposed, which is not observed in the real material.

In this section, we have demonstrated the possibility of additional ordering in *β*-Nb_3_Cl_8_ arising from the internal structure of the Nb_3_ unit. Since the amplitude of the scissoring distortion is dependent on the value of *U*_at_, and given that intertrimer bonding components become more relevant for other members of the Nb_3_*X*_8_ family, the approximation of being deep in the Mott state may break down in those cases. We would then expect this scissoring tendency to be less pronounced across the rest of the series, which aligns with experimental observations, having previously found the *C*2/*m* phase only in Nb_3_Cl_8_. Hence, we now turn to our experimental results for Nb_3_Cl_8_ and the mixed Nb_3_Cl_4_Br_4_ compound.

### Experimental

In order to validate the results of our computational investigation of Nb_3_Cl_8_ and to attempt to understand the origins of the differing LT phases reported in the literature, we have synthesized Nb_3_Cl_8_ and Nb_3_Cl_4_Br_4_ and characterized their crystallographic structure and electronic properties. Our samples of Nb_3_Cl_8_ were determined by single-crystal XRD to adopt the HT $P\bar{3}m1$ phase at 100 K, the lowest temperature at which we could perform these measurements. Magnetic susceptibility measurements (see Supplementary Fig. 7) on a single crystal show the phase transition to occur below 100 K on cooling and slightly above 100 K on heating, and our finding of the HT phase at 100 K is therefore ascribed to hysteresis of the phase transition.

**Fig. 6:**
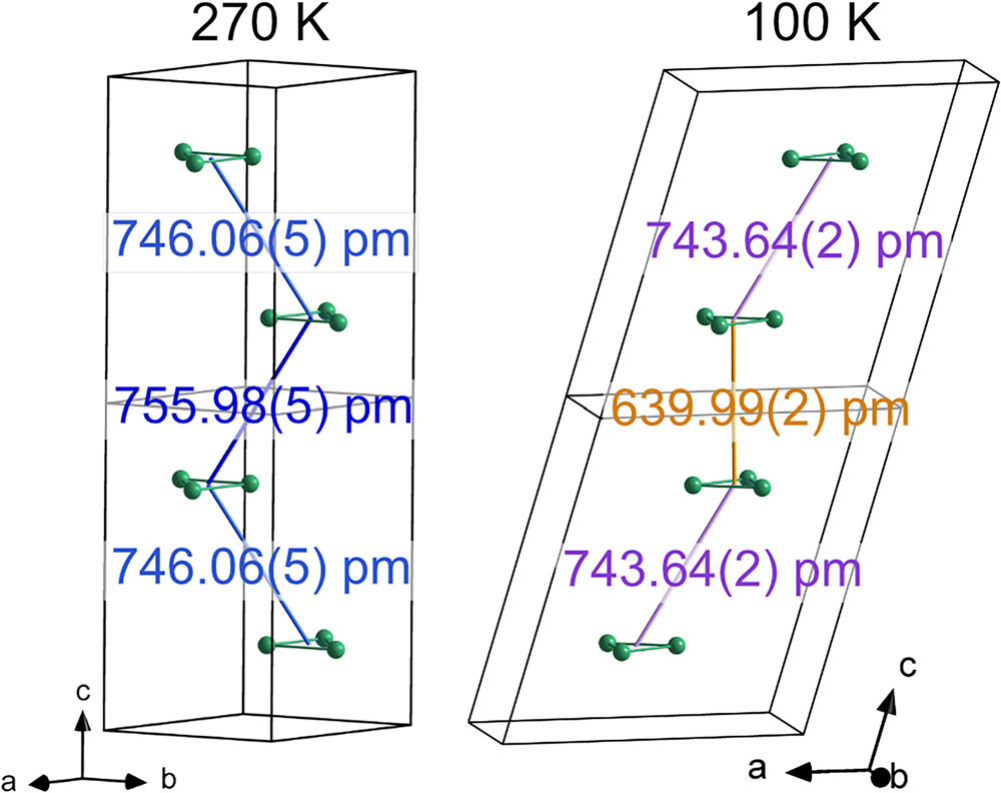
Stacking arrangement and distances of Nb_3_ clusters (green triangles) in Nb_3_Br_4−*x*_Cl_4+*x*_ as determined from single crystal X-ray diffraction data at 270 K (left) and 100 K (right). Chloride and bromide ions are omitted for clarity.

Therefore, in order to observe the LT phase, we prepared Nb_3_Br_4−*x*_Cl_4+*x*_, which was determined to have an increased phase transition temperature by Pasco et al.^29^. However, whereas the authors observed a LT $R\bar{3}m$ phase, we found the LT structure to adopt *C*2/*m* (Table 1, Fig. 6), indicating that the space group of LT Nb_3_Cl_4_Br_4_, like that of LT Nb_3_Cl_8_, possesses some ambiguity.

**Table 1:** Selected crystallographic data of Nb_3_Br_4−*x*_Cl_4+*x*_ above and below the transition temperature

Formula^a^	Nb_3_Br_3.7_Cl_4.3_	Nb_3_Br_3.8_Cl_4.2_
CCDC number	2364410	2365009
Temperature (K)	270.0(1)	100.0(1)
Crystal system	Trigonal	Monoclinic
Space group	$P\bar{3}m$	*C*2/*m*
Wavelength (pm)	154.184	71.037
Radiation type	Cu-K_*α*_	Mo-K_*α*_
Residual factor (*R*_1_)	0.0350	0.0350
Residual factor (*wR*_2_)	0.0903	0.0909

^a^The composition variation is within the synthesis and refinement error margin, and is considered as Nb_3_Br_4_Cl_4_.

Notably, our synthetic method, which employed only elemental Nb, NbCl_5_ and NbBr_5_ as reagents, follows that of Sheckelton et al.^30^, who found the *C*2/*m* phase in Nb_3_Cl_8_ at low temperatures. In contrast, synthesis using a transport agent such as NH_4_Cl or TeCl_4_ was reported to yield an LT $R\bar{3}m$ phase^29,31^, whereas reports of the *R*3 phase are associated with flux growth in PbCl_3_ followed by soaking in hot water^27,33^. These associations suggest that the precise LT phase adopted by Nb_3_Cl_8_ and Nb_3_Br_4−*x*_Cl_4+*x*_ could be determined by the synthetic conditions. In particular, tellurium has been reported to incorporate itself into the Nb_3_Cl_8_ structure by capping Nb_3_ triangles^48^, and the introduction of defects has been suggested to trap Nb_3_Br_8_ in its HT phase^29^; a similar effect related to the introduction of impurities from the transport agent could push the material into the $R\bar{3}m$ phase, which is more closely related to the HT $P\bar{3}m1$ phase than the *C*2/*m* is.

The changes between trimers in adjacent layers in Nb_3_Cl_4_Br_4_ following the phase transition can be readily seen in the single-crystal XRD refinement. At 270 K, the intertrimer distances are at 755.98(5) and 746.06(5) pm (Fig. 6, left). In the low-temperature structure (at 100 K), these distances alternate between 639.99(2) and 743.64(2) pm (Fig. 6, right), leading to the stacking shift (Fig. 1b) which modifies the intercluster interactions (Fig. 1c).

**Fig. 7:**
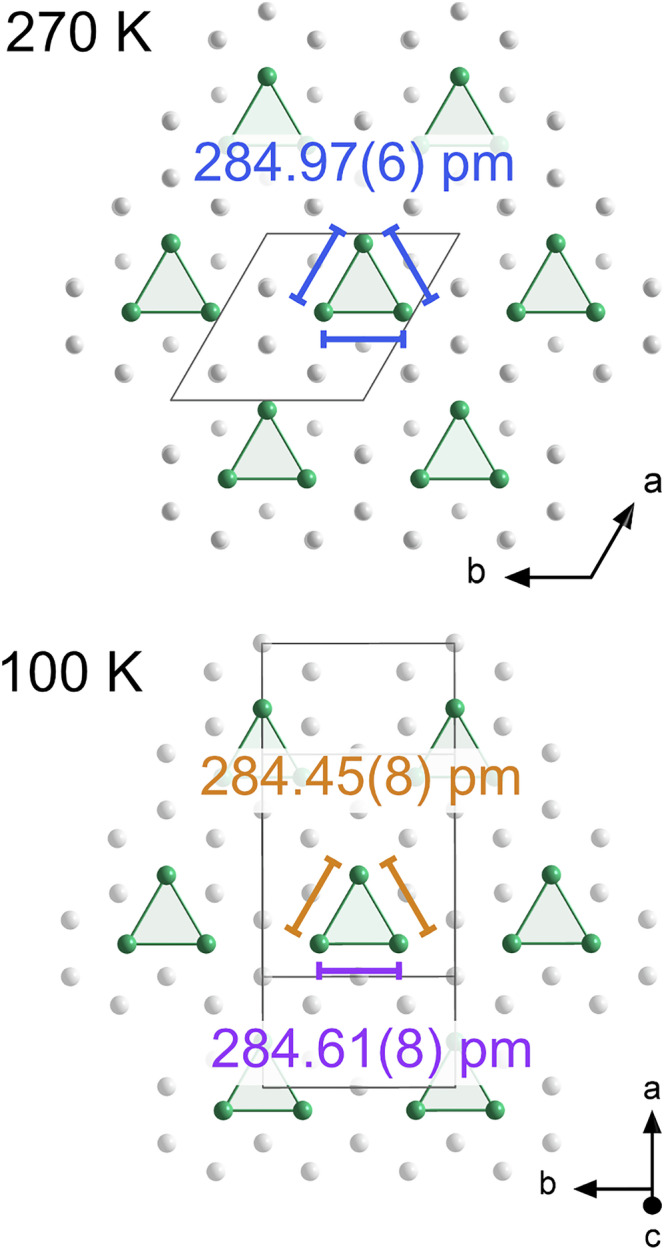
Nb–Nb distances within Nb_3_ clusters in Nb_3_Br_4−*x*_Cl_4+*x*_ as determined from single crystal X-ray diffraction data at 270 K (top) and 100 K (bottom). Niobium atoms are depicted in green, chloride and bromide ions are in gray. The unit cell vectors are shown as gray lines.

At 270 K, in the $P\bar{3}m1$ phase, the Nb–Nb distances within a cluster form equilateral triangles with bond lengths of 284.97(6) pm (Fig. 7, top). In the low-temperature *C*2/*m* phase at 100 K, the clusters adopt an isosceles triangular arrangement, with one Nb–Nb bond measuring 284.61(8) pm and the other two at 284.45(8) pm (Fig. 7, bottom). The asymmetry arises due to the unambiguous loss of 3-fold rotational symmetry upon cooling, but the overall change in bond lengths remains very small, to the point where one short and two long Nb–Nb bonds are possible within the experimental uncertainty. The difference in Nb–Nb bond lengths is much smaller in Nb_3_Cl_4_Br_4_ than was reported by Sheckelton et al. in Nb_3_Cl_8_^30^. According to our computational results discussed in the previous section, the change in the stacking sequence observed in Fig. 6 is driven by intertrimer interaction, while the symmetry reduction to *C*2/*m*, which removes the 3-fold rotation axis and allows the Nb–Nb bond lengths to differ, is likely a secondary effect driven by intratrimer interactions.

We conducted magnetic measurements on Nb_3_Cl_8_ to observe the phase transition, as this occurred below the minimum temperature at which we could perform crystallographic measurements. We see a similar phase transition temperature (ca. 100 K) and degree of hysteresis (see Supplementary Fig. 7) to previous reports of single crystals of Nb_3_Cl_8_^27,30,31^. However, unlike Haraguchi et al.^27^, who observed this transition only in single crystals, we were able to detect a magnetic phase transition in both single crystal and powder samples (see Supplementary Fig. 8). In comparison to the single crystal sample, the powder sample displays an increased hysteresis, and a stronger temperature dependence of the paramagnetic response below the transition temperature. This temperature dependence leads to suppression of the paramagnetism near the transition temperature upon heating, a phenomenon also observed in assemblages of single crystals^29^, suggesting that inter-grain interactions affect the magnetic properties of Nb_3_Cl_8_.

Optical measurements were performed above the structural transition temperature at room temperature. For both Nb_3_Cl_8_ and Nb_3_Cl_4_Br_4_, we observe similar direct and indirect optical band gaps (see Table 2), based on measurements on bulk samples consisting of small crystallites. This result indicates similar electronic properties of Nb_3_Cl_8_ and Nb_3_Cl_4_Br_4_, validating our use of Nb_3_Cl_4_Br_4_ as a proxy for low-temperature structural measurements. The values are consistent with literature reports^12,49^, but are smaller compared to our DFT + DFMT results.

**Table 2:** Direct and indirect optical band gaps for Nb_3_Cl_8_ and Nb_3_Br_4_Cl_4_ at room temperature, determined by DRIFT spectroscopy (see Supplementary Figs. 4 and 5 for full optical spectra)

Nb_3_Cl_8_	Direct	0.510 eV
	Indirect	0.344 eV
Nb_3_Br_4_Cl_4_	Direct	0.537 eV
	Indirect	0.311 eV

We investigated the temperature-dependent conductivity of bulk crystals and observed semiconducting behavior (see Supplementary Fig. 6) for the high temperature modification above 100 K. The conductivity ranges from ~2 × 10^−3^ S m^−1^ at 300 K to 8 × 10^−8^ S m^−1^ at 100 K. Below 100 K, the conductivity fell below the detection limit. No hysteresis was observed during cooling and heating cycles, which is consistent with the measurements being conducted above the structural transition temperature. As the magnetic susceptibility shows some hysteresis above 100 K (see Supplementary Fig. 7), our experimental results suggest that the electrical conductivity is not strongly affected by the magnetic transition, further indicating that the magnetic transition is not driven by charge disproportionation. Our results align with the earlier measurements by Yoon et al.^12^ at higher temperatures.

## Discussion

In this work, we performed a DFT + *U* + *V*, DFT + DMFT, and experimental study of the bulk LT *β*-Nb_3_Cl_8_ phase. Particularly, we studied the different proposed mechanisms of the observed magnetostructural transition, with a focus on the effects of intra- and intertrimer Coulomb interactions.

We showed that it is the dimerization of the layers due to the change in stacking that is the primary driver of the magnetic transition in these compounds, and that the charge disproportionation model^27,33^ is not consistent with our calculations. In particular, through a DFT + DMFT analysis, we show that Nb_3_Cl_8_ behaves as a system of weakly-coupled Hubbard dimers, and that two of the proposed explanations for the physics of Nb_3_Cl_8_, the Mott-like strongly antiferromagnetically coupled system, and the singlet-like band insulator, are smoothly connected, with the intertrimer interaction dictating their crossover. Additionally, the analysis of the intratrimer behavior suggests a possible mechanism for the experimentally observed scissoring of the trimer units.

The important role of the intertrimer interaction manifests itself also in the other members of the Nb_3_*X*_8_ family [see Supplementary Table 2] where a smaller *U*/*t* ratio leads to a larger proximity to the band insulator solution rather than a Mott-like behavior.

In line with the theoretical findings, single-crystal X-ray diffraction reveals a structural phase transition in Nb_3_Br_4_Cl_4_ from $P\bar{3}m1$ to *C*2/*m*, accompanied by a pronounced reduction in the distances between adjacent clusters. The bond lengths within the Nb_3_ clusters slightly decrease upon cooling across the transition. Symmetry breaking leads to a slight distortion of the equilateral Nb_3_ triangles into isosceles ones. However, the difference between the shorter and longer Nb–Nb bond lengths remains small and experimentally does not indicate a clear preference regarding whether the one inequivalent bond is longer or shorter than the others. The primary structural effect detected by our X-ray diffraction study is therefore a rearrangement of clusters of adjacent layers relative to one another, rather than significant changes within the clusters themselves. Magnetic measurements on Nb_3_Cl_8_ confirm the expected magnetic-to-nonmagnetic transition around 90 K, with clear hysteresis observed in both single crystals and powder samples. Temperature-dependent conductivity measurements showed a gradual decrease upon cooling to the transition temperature, without significant hysteresis. Below 100 K, no current could be detected within the sensitivity of the measurement setup. The differing hysteresis behavior of the magnetic and electric properties is consistent with our computational results, in particular, the absence of interlayer charge disproportionation.

In conclusion, our work provides further evidence that the magnetostructural transition in LT *β*-Nb_3_Cl_8_ is driven primarily by a change in crystal stacking to the $R\bar{3}m$ phase. The physics of this interlayer dimerization can be understood in terms of weakly coupled Hubbard dimers formed from pairs of Nb trimers. The subtle competition between intratrimer and intertrimer interactions tunes the system across a crossover between the limits of a Mott insulator and a band insulator. Concurrently, Coulomb interactions within a trimer drive a secondary, more subtle scissoring distortion within the Nb_3_ trimer units.

This mechanism highlights the general importance of interlayer coupling in determining the electronic and magnetic properties of the Nb_3_*X*_8_ family of materials. Our calculations suggest that targeted control of external parameters such as pressure, chemical substitution, or strain might allow systematic exploration of the crossover from Mott to band insulator and engineering of new functionalities in this and related cluster Mott systems.

## Methods

### DFT + *U* + *V* calculations

We perform DFT + *U* + *V* calculations using the QUANTUM ESPRESSO package (v6.6)^47,50^ within the generalized gradient approximation with the Perdew–Burke–Ernzerhof^51^ exchange-correlation functional, utilizing the semiempirical DFT-D3 correction^52^ to account for the van der Waals effects. We use the scalar-relativistic ultrasoft pseudopotentials from the GBRV library^53^ with semicore 4*s* and 4*p* states included as valence for the Nb atoms.

We construct a localized set of orbitals using WANNIER90 (v3.1.0)^54,55^. Starting from conventional non-spin-polarized DFT calculations, we consider the two bands in close proximity to the Fermi level with dominant Nb contributions and with no entanglement with the surrounding bands (see Fig. 1e). Following Grytsiuk et al.^20^, our basis set consists of two trimer-centered Wannier functions computed by projecting the Kohn–Sham (KS) states around the Fermi level onto initial guesses of ${d}_{{z}^{2}}$ orbitals and performing subsequent orthonormalization to finally obtain the molecular trimer orbitals shown in Fig. 1f. All structural and Wannier function visualizations were produced using VESTA^56^.

We then employ these trimer orbitals in both spin-unpolarized and spin-polarized DFT + *U* + *V* calculations using the implementation from ref. ^41^. We consider both onsite (intratrimer) and intersite (intertrimer) interactions described by parameters *U*_tr_ and *V*_tr_, respectively (see Fig. 1c).

When performing structural variation, the Wannier functions are recalculated at every step.

### DFT + DMFT calculations

The trimer basis set for the DFT + DFMT calculations is described by the same Wannier functions obtained in the previous section. We consider an impurity problem composed of two adjacent trimer orbitals, a “dimer of trimers”, for which the Hamiltonian reads:

5 $$H={H}_{0}+{H}_{{\rm{int}}}-{H}_{{\rm{DC}}},$$

where *H*_0_ corresponds to the effective single-particle DFT Hamiltonian as obtained in the wannierization procedure and *H*_int_ is the interaction Hamiltonian:

6 $${H}_{\mathrm{int}}={U}_{\mathrm{tr}}{\mathop{\sum}\limits_{i}}\hat{n}_{i}^{\uparrow }{\hat{n}}_{i}^{\downarrow }+{V}_{\mathrm{tr}}\mathop{\sum }\limits_{\langle ij\rangle ,\sigma {\sigma }^{{\prime} }}{\hat{n}}_{i}^{\sigma }{\hat{n}}_{j}^{\sigma {\prime} },$$

where ${\widehat{n}}_{i}^{\sigma }$ is the spin resolved number operator for trimer site *i* and spin *σ*. The meaning of *U*_tr_ and *V*_tr_ corresponds to the same as in the DFT + *U* + *V* case, that of an onsite (intratrimer) and intersite (intertrimer) interaction, respectively.

*H*_DC_ is the double-counting (DC) term, which cancels out the part of local interaction already included in the *H*_0_ term^39^. Here, we employ the standard fully localized limit for the expression of the energy of the double counting correction^57^, which in this basis set, translates to a constant shift of the trimer orbital energies:

7 $${\Sigma }_{\,{\rm{DC}}}^{tr}=U\mathop{\sum }\limits_{i,\sigma }\left({N}_{i}^{\,{\rm{tr}}}-\frac{1}{2}\right){\widehat{n}}_{i}^{\sigma }.$$

Here, ${N}_{i}^{\,{\rm{tr}}}={\sum }_{\sigma }\langle {n}_{i}^{\sigma }\rangle$ is the occupation of the trimer orbitals. In the presence of a difference in occupation between the two trimers, i.e., a charge disproportionation scenario, this term could, in principle, favor the localization of two electrons on one trimer. However, similarly to the DFT + *U* + *V* calculations, we find no trace of this tendency in our DFT + DMFT calculations, and both trimers remain equally occupied. We note that we have not included an intersite contribution to the double-counting potential. The approach outlined in ref. ^39^ and used in the DFT + *U* + *V* calculations, effectively removes the double counting of the Hartree contribution stemming from intersite interactions, which would further suppress charge-disproportionation in the present case. However, given that our DFT + DMFT calculations already show no tendency for charge order and maintain equally occupied trimers, we do not anticipate that including this term would have any effect on our results.

In the atomic basis, we construct Wannier functions, including all six bands in the energy range between −0.5 and 1.5 eV around the Fermi level. This results in one *d* orbital per Nb site oriented towards the center of the trimer. We then perform DFT + DMFT calculations with 2 impurity models, each one including the 3 atomic orbitals belonging to one Nb_3_ unit. The resulting Hamiltonian has the same form as described in Eq. (5), however, the *H*_0_ term this time represents the tight binding model described by the atomic Wannier basis set, and *H*_int_ reads:

8 $${H}_{\mathrm{int}}={U}_{\mathrm{at}}\mathop{\sum }\limits_{i}{\hat{m}}_{i}^{\uparrow }{\hat{m}}_{i}^{\downarrow }+{V}_{\,\mathrm{at}}^{\mathrm{intra}}\mathop{\sum }\limits_{\langle ij\rangle ,\sigma \sigma {\prime} }{\hat{m}}_{i}^{\sigma }{\hat{m}}_{j}^{\sigma {\prime} },$$

where now ${\widehat{m}}_{i}^{\sigma }$ is the number operator for an atomic orbital on Nb site *i* and spin channel sigma *σ*, and ${V}_{\,{\rm{at}}}^{{\rm{intra}}}$ is the Nb–Nb interaction inside one trimer. For the DC correction, we again employ the fully localized limit, but this time in the atomic basis:

9 $${\Sigma }_{\,\mathrm{DC}}^{\mathrm{at}}=\mathop{\sum }\limits_{i}U\left({M}_{i}^{\,\mathrm{at}}-\frac{1}{2}\right){\hat{m}}_{i}^{\sigma }\,,$$

where ${M}_{i}^{\,{\rm{at}}}={\sum }_{\sigma }\langle {m}_{i}^{\sigma }\rangle$. These terms translate to a downward energetic shift of the occupied atoms and an upward shift of the unoccupied ones.

In our DMFT calculations for the three-atom clusters, we have again omitted the intersite contribution to the double-counting corrections. Given that the material is deep in the Mott regime, the strong Coulomb interaction enforces a local filling of exactly one electron per Nb_3_ cluster. The subspace of relevant electronic configurations is therefore composed almost exclusively of states with a single occupied atomic orbital per trimer. Within this subspace, the Hamiltonian term for the intersite interaction, ${V}_{at}^{\,{\rm{intra}}}{\sum }_{ij}{m}_{i}^{\sigma }{m}_{j}^{\sigma {\prime} }$, always acts as the zero operator and, in fact, we performed benchmark calculations and we see a negligible effect of the value of ${V}_{\,{\rm{at}}}^{{\rm{intra}}}$. In this sense, since the ${V}_{\,{\rm{at}}}^{{\rm{intra}}}$ does not seem to contribute to the self-energy, we deem the introduction of an inter-atomic DC correction unnecessary.

All our DFT+DMFT calculations are fully charge self-consistent and were performed using solid_dmft^58^, which is part of the TRIQS library^59^. We solve all the DMFT impurity problems with the continuous-time quantum Monte Carlo solver CT-HYB^60–62^ at the inverse electronic temperature close to room temperature of $\beta ={({k}_{{\rm{B}}}T)}^{-1}=40\,{{\rm{eV}}}^{-1}$. We note that this is purely the electronic temperature of the chosen solver. We use 10^4^ warm-up steps and 2 × 10^8^ Monte Carlo cycles with 120 steps each. We average over both spin channels to ensure a paramagnetic solution.

From the local Green’s function, ${G}_{\nu \nu {\prime} }(\tau )$, where *ν*, $\nu {\prime}$ correspond to different orbitals and *τ* is the imaginary time, we obtain the local occupations on a given site (trimer), ${n}_{\nu \nu {\prime} }={G}_{\nu \nu {\prime} }(\tau ={0}^{-})$, as well as the averaged spectral weight around the Fermi level, $\bar{A}(\omega =0)=-(\beta /\pi )\,\mathrm{Tr}\,G(\tau =\beta /2)$. Additionally, we calculate the quasiparticle weight, $Z={[1-{\left.\partial {\rm{Im}}\Sigma (i\omega )/\partial (i\omega )\right|}_{i\omega \to 0}]}^{-1}$, from the imaginary part of the local self-energy, *Σ*, by fitting a third-order polynomial to the lowest five Matsubara frequencies and interpolating to zero frequency. Finally, we use the maximum-entropy method^63,64^ to obtain the *k*-averaged spectral functions on the real frequency axis.

### cRPA calculations

For the cRPA calculations, norm-conserving pseudopotentials from PSEUDODOJO^65,66^ are used to perform the initial DFT calculations. For these, we use a kinetic energy cutoff of 52 Ry (~700 eV) and 12 × 52 Ry for the charge density. We converged the total energies to 10^−3^ Ry using a 6 × 6 × 3 *k*-point, smearing the occupations with the Marzari–Vanderbilt scheme^67^ with a smearing parameter of 0.001 Ry.

Screened Coulomb interaction parameters are obtained using the cRPA code RESPACK^68^, using the WAN2RESPACK interface to QUANTUM ESPRESSO^69^. We set the polarization function cutoff to 15 Ry, and we use a total of 300 bands, setting the upper limit of the highest possible excitations to 25 eV above the Fermi energy.

### Experimental details

For the synthesis of Nb_3_Cl_8_, a mixture of NbCl_5_ (76.9 mg, 28.4 mmol ABCR GmbH, 99.9%) and Nb (23.1 mg, 24.9 mmol, ABCR GmbH, 99.9%), fused into an evacuated silica tube, was heated with 0.5 K/min in a Simon–Müller furnace. After heating at 750 ^∘^C for 48 h, Nb_3_Cl_8_ was obtained as plate-like crystals (yield: >95%).

For the synthesis of Nb_3_Br_4_Cl_4_, a mixture of NbBr_5_ (79.8 mg, 16.2 mmol, ABCR GmbH, 99.9%), NbCl_5_ (43.8 mg, 16.2 mmol ABCR GmbH, 99.9%), and Nb (26.4 mg, 28.4 mmol, ABCR GmbH, 99.9%), fused into an evacuated silica tube, was heated with 0.5 K/min in a Simon–Müller furnace. After heating at 750 ^∘^C for 48 h, Nb_3_Br_4_Cl_4_ was obtained as plate-like crystals (yield: >90%).

Single-crystal X-ray diffraction was used for structure characterization. To maintain crystal integrity, separate single crystals were selected for each measurement and gradually cooled to the target temperature at a rate of 2 K/min. Data collection was performed on a Rigaku XtaLAB Synergy-S single-crystal X-ray diffractometer equipped with a HyPix-6000HE detector and monochromated Mo-K_*α*_ radiation (*λ* = 71.037 pm) at 100 K and monocromated Cu-K_*α*_ radiation (*λ* = 154.184 pm) at 270 K. X-ray intensities were corrected for absorption with a numerical method (crystal faces) using CrysAlisPro 1.171.43.121a (Rigaku Oxford Diffraction, 2024). Structures were solved by direct methods (SHELXT) and refined by full-matrix least squares methods performed with SHELXL-2019/3^70^ as implemented in Olex2 1.5^71^. Detailed crystallographic data can be obtained free of charge via www.ccdc.cam.ac.uk and the CCDC numbers.

Electrical conductivity measurements were performed in a Lake Shore Cryotronics CRX-6.5K probe station with a Keithley 2636B source meter unit. Plate-like crystals of Nb_3_Cl_8_ were transferred into the chamber under protective gas and contacted with silver paste on a silicon substrate with a 770 nm oxide layer. The conductive silver pads at each end of the crystals were connected to the circuit with gold-coated tungsten tips. The chamber was kept under vacuum (>5 × 10^−5^ mbar) and the temperature was varied between 20 and 300 K (see Supplementary Fig. 6). Before each measurement, sufficient time was allowed for the sample to reach the chosen temperature. Two-point conductivity measurements were performed by varying the applied source–drain voltage from −1 to 1 V while detecting the current.

Diffuse reflectance infrared Fourier transformation spectroscopy (DRIFT) samples were measured at room temperature under inert conditions in diffuse reflectance with a Harrick Praying Mantis attachment using a Bruker Vertex 70 infrared spectrophotometer with a deuterated triglycine sulfate (DTGS) detector and KBr beamsplitter. The background spectra were collected using pure dried KI in powder form.

Magnetic susceptibility measurements of crystalline powders and single crystals were performed in gelatine capsules between 5 and 300 K with a Quantum Design SQUID magnetometer equipped with a 1802 R/G bridge and a 1822 MDMS controller.

## Supplementary information

Supplementary information

## Data Availability

The data from the DFT + *U* + *V* and DFT + DMFT calculations presented in this study are available at https://zenodo.org/records/17898535.
