# Supplementary material for: Hubbard dimer physics and the magnetostructural transition in the correlated cluster material Nb3Cl8
Source: NPJ 2D Mater Appl. 2026 May 20;10(1):74. doi: 10.1038/s41699-026-00706-0 (PMC13461348; doi:10.1038/s41699-026-00706-0)
Supplement: Supplementary file 1 [file 41699_2026_706_MOESM1_ESM.pdf]

Supplementary Information for "Hubbard dimer physics and the magnetostructural transition in the correlated cluster material Nb_3Cl_8"

Alberto Carta,[*,1,2,5] Peter Mlkvik,[*,2,5] Fabian Grahlow,[3] Markus Ströbele,[3] H.-Jürgen Meyer,[3] Carl P. Romao,[4] Nicola A. Spaldin,[2] and Claude Ederer[2]

[1]*PSI Center for Scientific Computing, Theory, and Data, 5232 Villigen PSI, Switzerland*

[2]*Materials Theory, ETH Zürich, Wolfgang-Pauli-Strasse 27, 8093 Zürich, Switzerland*

[3]*Section for Solid State and Theoretical Inorganic Chemistry, Institute of Inorganic Chemistry, Eberhard-Karls-Universität Tübingen, Auf der Morgenstelle 18, 72076 Tübingen, Germany*

[4]*Department of Materials, Faculty of Nuclear Sciences and Physical Engineering, Czech Technical University in Prague, Trojanova 13, Prague 120 00, Czech Republic*

[5]*These authors contributed equally to this work.*

*E-mail: alberto.carta@psi.ch; peter.mlkvik@mat.ethz.ch

Model parameters for the Nb_3X_8 family

The structural parameters calculated in the first-principles calculations, such as the a and c lattice parameters of the unit cells used, the small (d_{LT}) and large (d'_{LT}) interlayer intertrimer distances, as shown in Fig. 1(b) of the original manuscript, and the intratrimer interatomic ($d_{\mathrm{Nb-Nb}}$) distances for Nb_3Cl_8, Nb_3Br_8, and Nb_3I_8 are all given in Supplementary Table 1 for the $R\bar{3}m$ LT structures. The size of the unit cells increases through the series, as do the intertrimer distances.

In Supplementary Table 2, we give the large (t_{LT}) and small (t'_{LT}) interlayer intertrimer hopping parameters, as shown in Fig. 1(c) of the original manuscript, as well as the intralayer intertrimer hopping parameter ($t_{\mathrm{LT}}^{\mathrm{in-plane}}$). Additionally, we also give the calculated Coulomb interaction parameters using cRPA [1, 2], where we give both the bare unscreened ($\widetilde{U}_{\mathrm{tr}}$, $\widetilde{V}_{\mathrm{tr}}$, $\widetilde{V}_{\mathrm{tr}}^{\mathrm{in-plane}}$), and screened ($U_{\mathrm{tr}}$, V_{tr}, $V_{\mathrm{tr}}^{\mathrm{in-plane}}$) onsite, interlayer intertrimer, and intralayer intertrimer interaction parameters, respectively, as shown in Fig. 1(c) of the original manuscript. We give results for Nb_3Cl_8, Nb_3Br_8, and Nb_3I_8 in the $R\bar{3}m$ LT structures as specified in Supplementary Table 1.

In the trimer basis, the larger interlayer intertrimer hopping amplitude (corresponding to the "dimerized trimer") clearly dominates over the smaller interlayer intertrimer and the in-plane hopping amplitudes. These change monotonically across the series, with the hopping strength increasing with increasing atomic number. The values of U_{tr} and V_{tr} also change monotonically throughout the series, with increasing $V_{\mathrm{tr}}/U_{\mathrm{tr}}$ ratio from 1/4 to approximately 1/3. Both the U and the V parameters are strongly screened with increasing ratio of screening ($U/\widetilde{U}$) across the compounds – 76% to 83% screening for U. The in-plane interaction strength is comparable to that out of plane.

In Supplementary Table 3, we show the results for the atomic basis, namely the intratrimer (t_{intra}) and largest intertrimer (t_{inter}) (inside the "dimerized trimers") hopping parameters, the bare ($\widetilde{U}_{\mathrm{at}}$, $\widetilde{V}_{\mathrm{at}}^{\mathrm{intra}}$, $\widetilde{V}_{\mathrm{at}}^{\mathrm{inter}}$), and screened ($U_{\mathrm{at}}$, $V_{\mathrm{at}}^{\mathrm{intra}}$, $V_{\mathrm{at}}^{\mathrm{inter}}$) onsite and intratrimer interatomic and largest intertrimer interlayer interatomic interaction parameters,

respectively, as obtained from cRPA for $R\bar{3}m$ LT structures of Nb_3Cl_8, Nb_3Br_8, and Nb_3I_8.

Similarly to the trimer basis, U_{at} and V_{at} decrease monotonically across the entire series. Notably, the intertrimer $V_{\mathrm{at}}^{\mathrm{inter}}$ interaction remains quite large in the atomic basis, being practically identical to the V_{tr} values reported in Supplementary Table 2 for the trimer basis.

Our hopping amplitudes and cRPA results for the trimer basis show good agreement with previous works [3, 4] performed for the $R\bar{3}m$ and $R3$ structures. As expected, since the underlying structures do not differ except for a slight symmetry-breaking, the results remain comparable. Calculations for the atomic basis have so far been only performed in the monolayer [3], where all the interaction parameters are understandably larger than the ones shown here due to the lack of screening in a 2D structure.

Supplementary Table 1: The a and c lattice parameters, the small (d_{LT}) and large (d'_{LT}) interlayer intertrimer distance, and the intratrimer interatomic ($d_{\mathrm{Nb-Nb}}$) distances for Nb_3Cl_8, Nb_3Br_8, and Nb_3I_8. All results for the $R\bar{3}m$ LT structures, all numbers given in Å.

(in Å)	Nb_3Cl_8	Nb_3Br_8	Nb_3I_8
a	5.86	6.16	6.61
c	12.38	13.16	14.14
d_{LT}	6.13	6.61	7.10
d'_{LT}	7.25	7.73	8.31
$d_{\mathrm{Nb-Nb}}$	2.81	2.87	3.00

In Supplementary Fig. 2, we show the evolution of the energy difference between the nominal Nb^{2+} and Nb^{3+} local onsite atomic energy levels that is induced by the scissoring distortion within a trimer, as discussed in text and in Fig. 5 of the original manuscript. The behavior is largely linear, with the experimentally observed scissoring distortion from Ref. [6] causing a moderately large splitting of around 0.12 eV.

In Supplementary Fig. 3 we show the off-diagonal occupation in analogy to Fig. 4(c) of the original manuscript, indicating the positions of the cRPA-calculated interaction parameters

Supplementary Table 2: The large (t_{LT}) and small (t'_{LT}) interlayer intertrimer hopping parameters, the intralayer intertrimer hopping parameter ($t_{LT}^{in-plane}$), the bare ($\widetilde{U}_{tr}$, $\widetilde{V}_{tr}$, $\widetilde{V}_{tr}^{in-plane}$), and screened ($U_{tr}$, V_{tr}, $V_{tr}^{in-plane}$) onsite, interlayer intertrimer, and intralayer intertrimer interaction parameters, respectively, as obtained from cRPA for Nb_3Cl_8, Nb_3Br_8, and Nb_3I_8. All results in the trimer basis and for the $R\bar{3}m$ LT structures, all numbers given in eV.

(in eV)	Nb_3Cl_8	Nb_3Br_8	Nb_3I_8
t_{LT}	-0.132	-0.153	-0.203
t'_{LT}	-0.004	-0.006	-0.010
$t_{LT}^{in-plane}$	0.020	0.005	-0.012
$\widetilde{U}_{tr}$	6.00	5.73	5.10
U_{tr}	1.46	1.21	0.85
$\widetilde{V}_{tr}$	2.20	2.11	1.99
V_{tr}	0.38	0.36	0.28
$\widetilde{V}_{tr}^{in-plane}$	2.06	1.94	1.80
$V_{tr}^{in-plane}$	0.45	0.37	0.28

Supplementary Table 3: The intratrimer (t_{intra}) and largest interplane intertrimer (t_{inter}) hopping parameters, the bare ($\widetilde{U}_{at}$, $\widetilde{V}_{at}^{intra}$, $\widetilde{V}_{at}^{inter}$), and screened ($U_{at}$, V_{at}^{intra}, V_{at}^{inter}) onsite and intratrimer interatomic and largest intertrimer interatomic interaction parameters, respectively, as obtained from cRPA for Nb_3Cl_8, Nb_3Br_8, and Nb_3I_8. All results in the atomic basis and for the $R\bar{3}m$ LT structures, all numbers given in eV.

(in eV)	Nb_3Cl_8	Nb_3Br_8	Nb_3I_8
t_{intra}	-0.313	-0.304	-0.268
t_{inter}	-0.045	-0.055	-0.074
$\widetilde{U}_{at}$	9.70	9.39	8.62
U_{at}	2.33	2.00	1.52
$\widetilde{V}_{at}^{intra}$	4.96	4.78	4.44
V_{at}^{intra}	1.36	1.10	0.78
$\widetilde{V}_{at}^{inter}$	2.02	1.98	1.87
V_{at}^{inter}	0.39	0.35	0.27

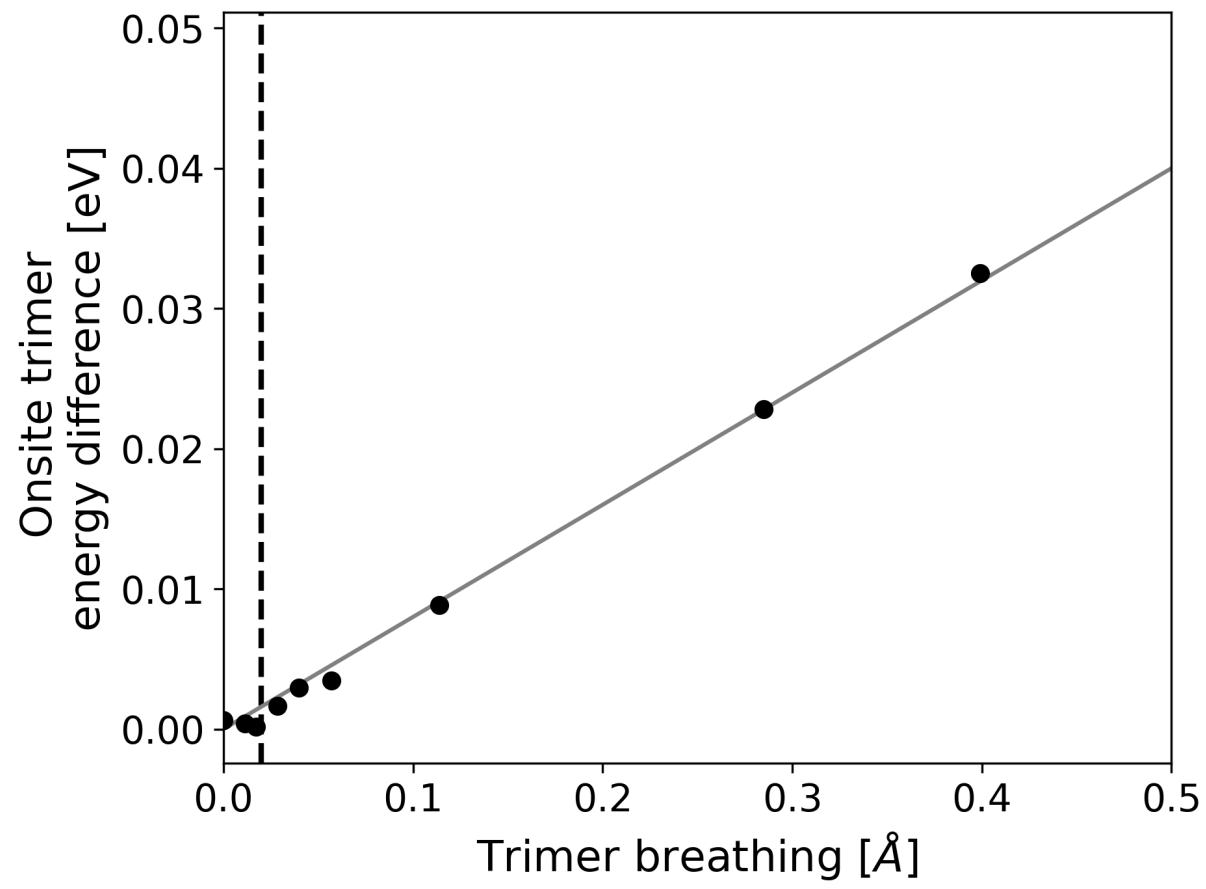

Supplementary Figure 1: The difference between the local onsite trimer energy levels [E(large trimer) $-$ E(small trimer)] as a function of the trimer breathing distortion, first shown in Fig. 2 of the original manuscript. The vertical dashed line indicates the experimentally reported amplitude of the breathing mode from Ref. [5].

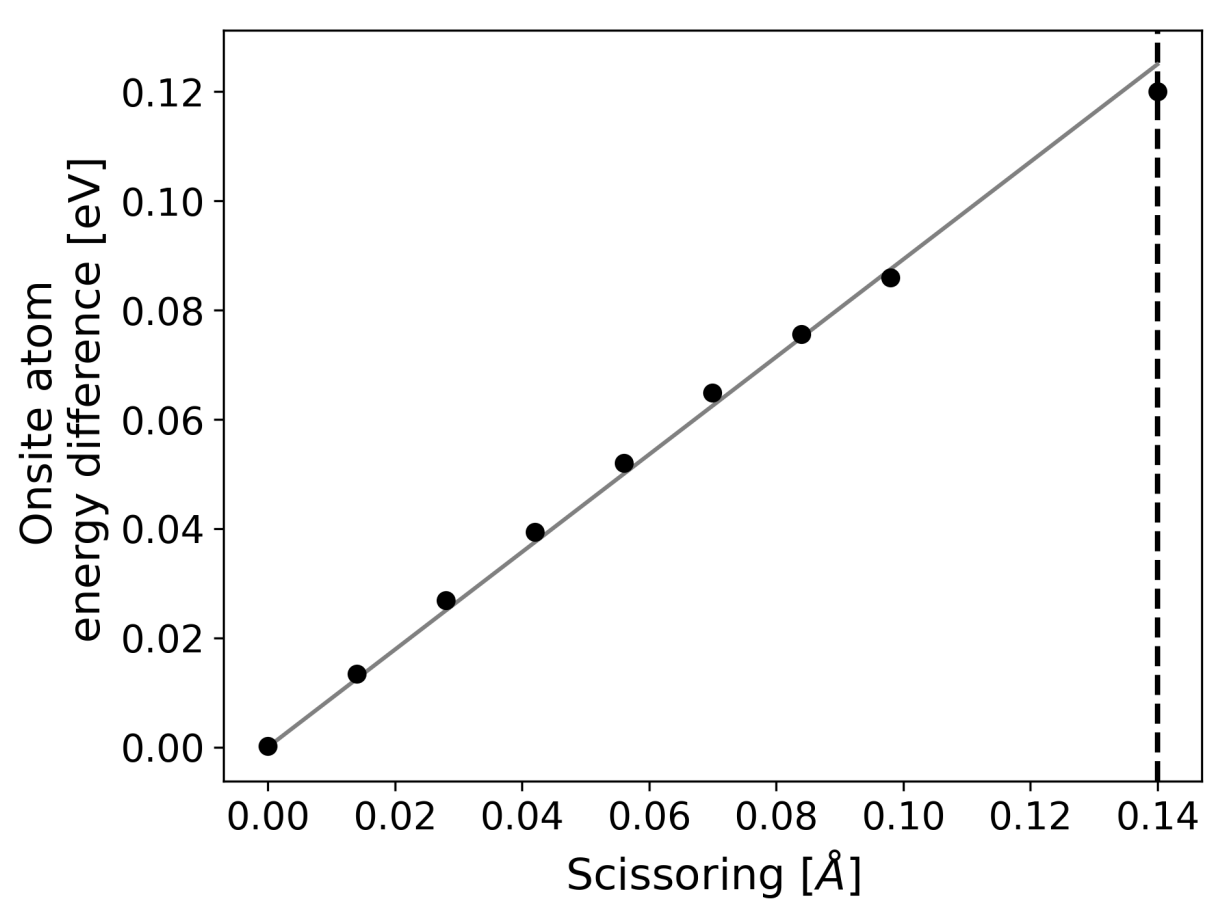

Supplementary Figure 2: The difference between the nominal Nb^{2+} and Nb^{3+} local onsite atomic energy levels as a function of the intratrimer scissoring distortion [$E(Nb^{2+}) - E(Nb^{3+})$], first shown in Fig. 5 of the original manuscript. The vertical dashed line corresponds to the experimentally reported scissoring amplitude from Ref. [6].

of the Nb_3X_8 compounds in the trimer basis from Supplementary Table 2 (shown as star, square, and triangle, respectively). As we move further down the series, the ratio of $U_{\rm tr}/V_{\rm tr}$ changes, and we get closer to the singlet insulating regime edge of the diagram, aided by the marked change in $t_{\rm LT}$ [not shown in Supplementary Fig. 3]. The increasing proximity to the band insulator is also the reason why the other compounds in the series, Nb_3Br_8 and Nb_3I_8, both feature a band gap already with conventional DFT.

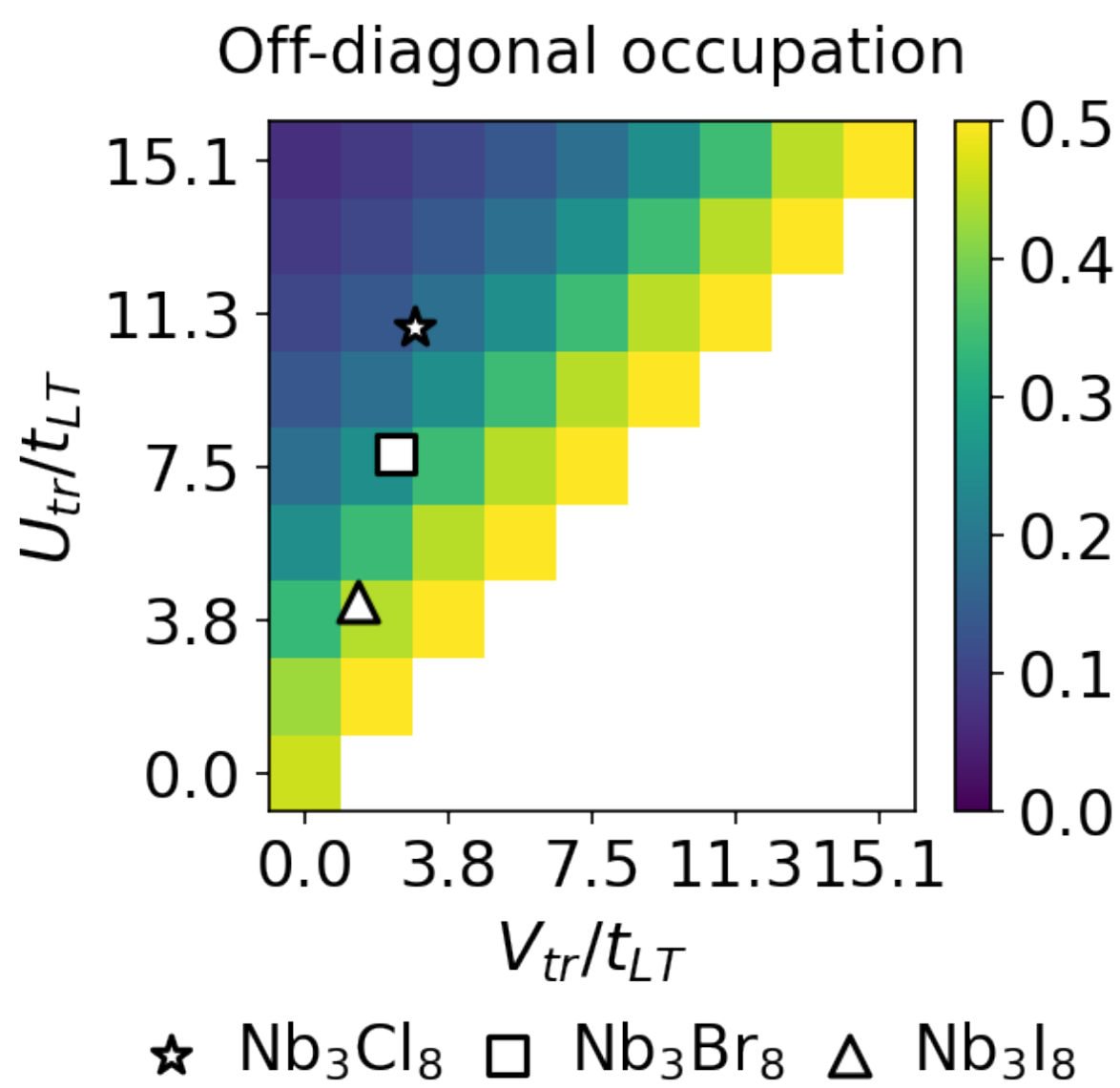

Supplementary Figure 3: The off-diagonal occupation of the Nb_3X_8 cluster from DFT+DMFT as a function of $U_{\rm tr}/t_{\rm LT}$ and $V_{\rm tr}/t_{\rm LT}$. The cRPA values for Nb_3Cl_8, Nb_3Br_8, and Nb_3I_8 are shown with a circle, square, and triangle, respectively.

Additional experimental data

Optical band gap determination from DRIFTS (diffuse reflectance infrared fourier transform spectroscopy)

The absorption coefficient F was obtained using Kubelka–Munk analysis following:

$$F(R_\infty) = \frac{\alpha}{S} = \frac{(1 - R_\infty)^2}{2R_\infty}, \tag{1}$$

where $R_\infty = \frac{R_{\text{sample}}}{R_{\text{standard}}}$ is the reflectance of an infinitely thick specimen, α is the absorption coefficient, and S is the scattering coefficient. For particle sizes greater than the light wavelengths measured, the scattering coefficient is understood to be approximately independent of frequency ($F(R_\infty) \sim \alpha$) and therefore $F(R_\infty)$ could be understood as a "pseudo-absorbanc" coefficient [7–9].

The band gap determination was performed on the DRIFTS data according to Zanata *et al.* [10]. The energy is plotted against the absorption coefficient α and fitted with a sigmoid-Boltzmann function:

$$\alpha(E) = \alpha_{\text{max}} + \frac{\alpha_{\text{min}} - \alpha_{\text{max}}}{1 + \exp\left(\frac{E - E_0^{\text{Boltz}}}{\delta E}\right)}, \tag{2}$$

where α_{min} (α_{max}) stands for the minimum (maximum) absorption coefficient; E_0^{Boltz} is the energy coordinate at which the absorption coefficient is halfway between α_{min} and α_{max}; and δE is associated with the slope of the sigmoid, indicating the energy range over which most optical transitions occur [10].

The band gap can then be calculated by the following equation with $n_{\text{dir}}^{\text{Boltz}} = 0.3$ and $n_{\text{indir}}^{\text{Boltz}} = 4.3$:

$$E_g^{\text{Boltz}} = E_0^{\text{Boltz}} - n_{(\text{dir/indir})}^{\text{Boltz}} \cdot \delta E. \tag{3}$$

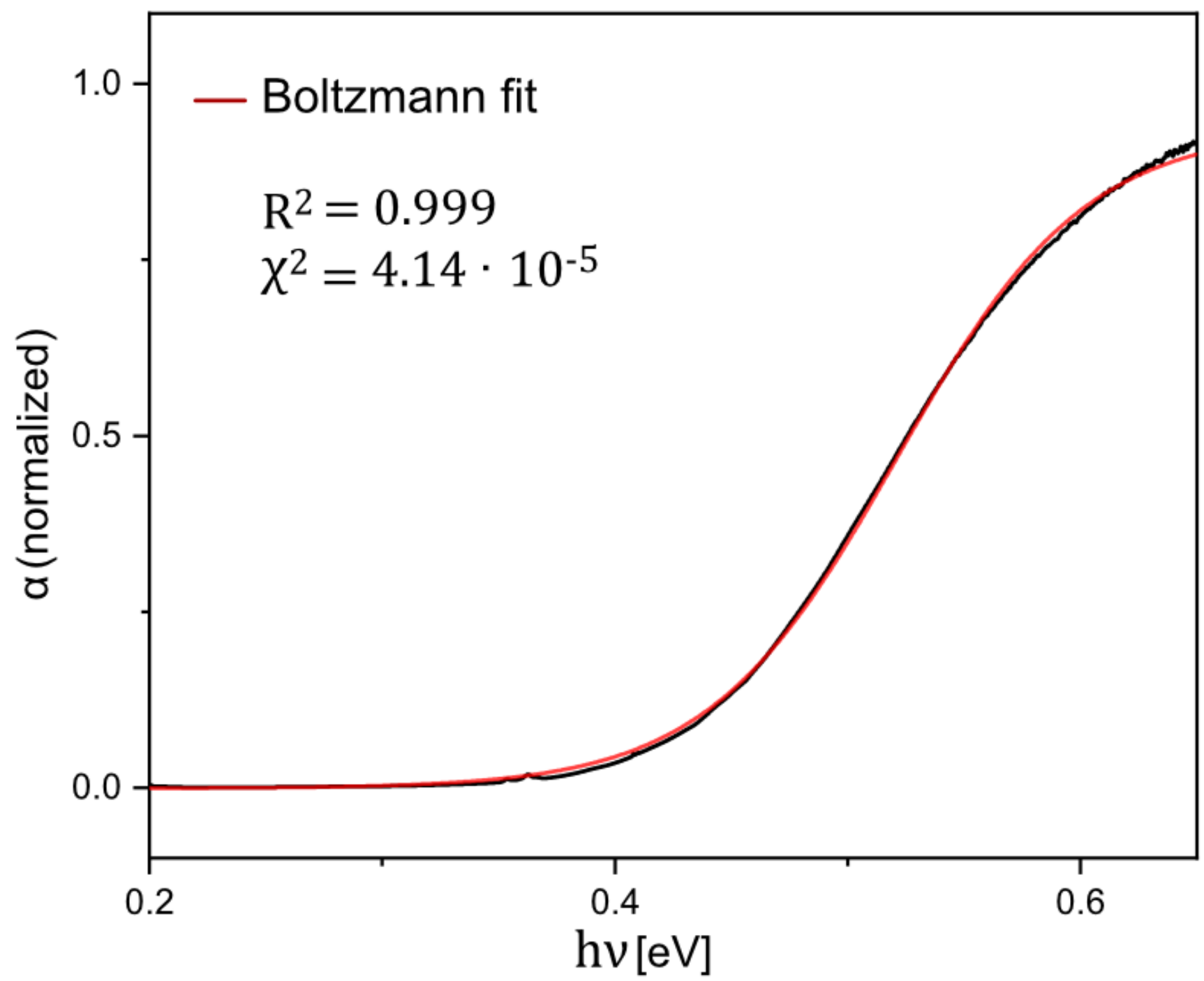

Supplementary Figure 4: Optical absorption coefficient spectra of Nb_3Cl_8 with the Boltzmann function used to fit α (normalized).

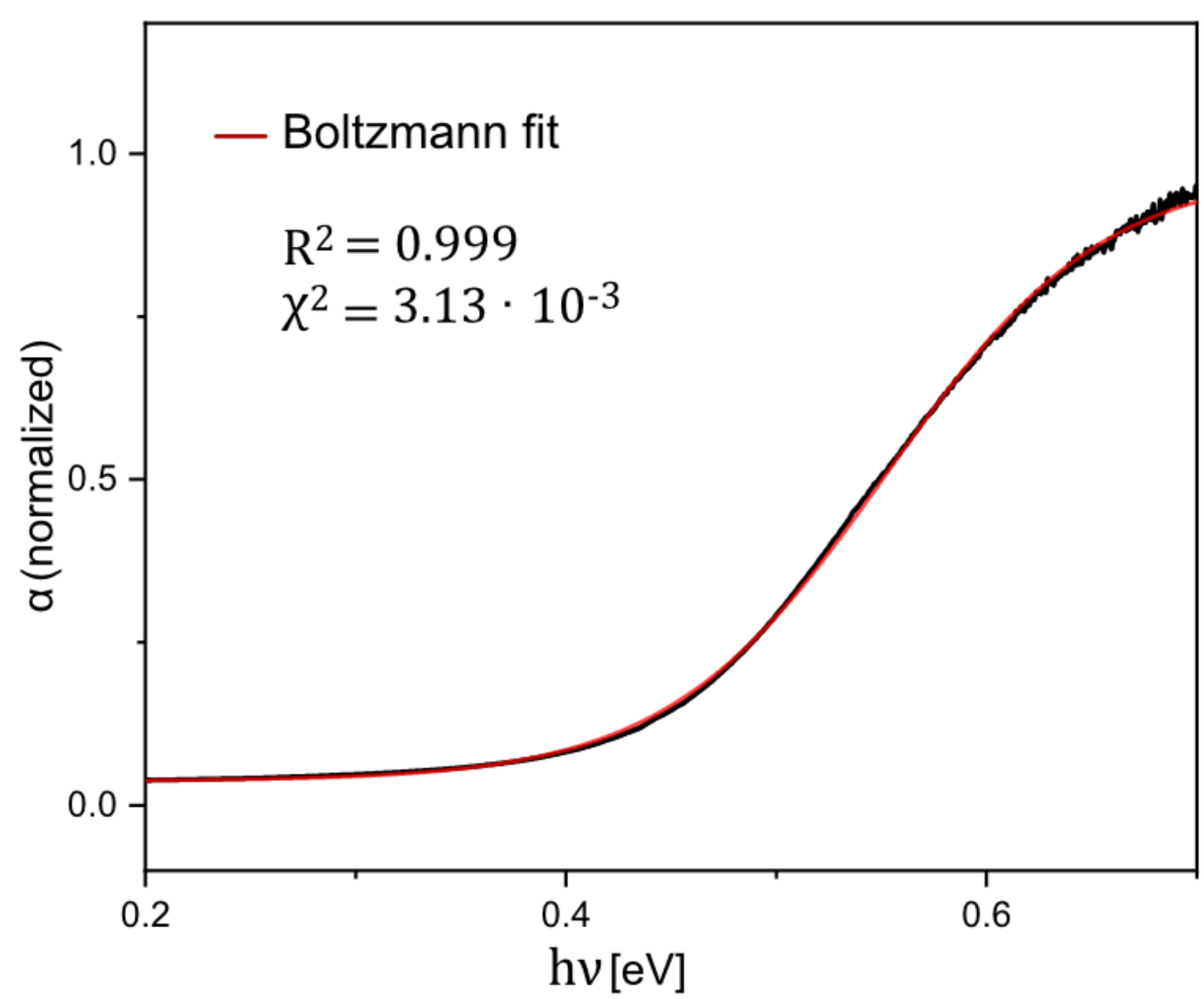

Supplementary Figure 5: Optical absorption coefficient spectra of $Nb_3Br_4Cl_4$ with the Boltzmann function used to fit α (normalized).

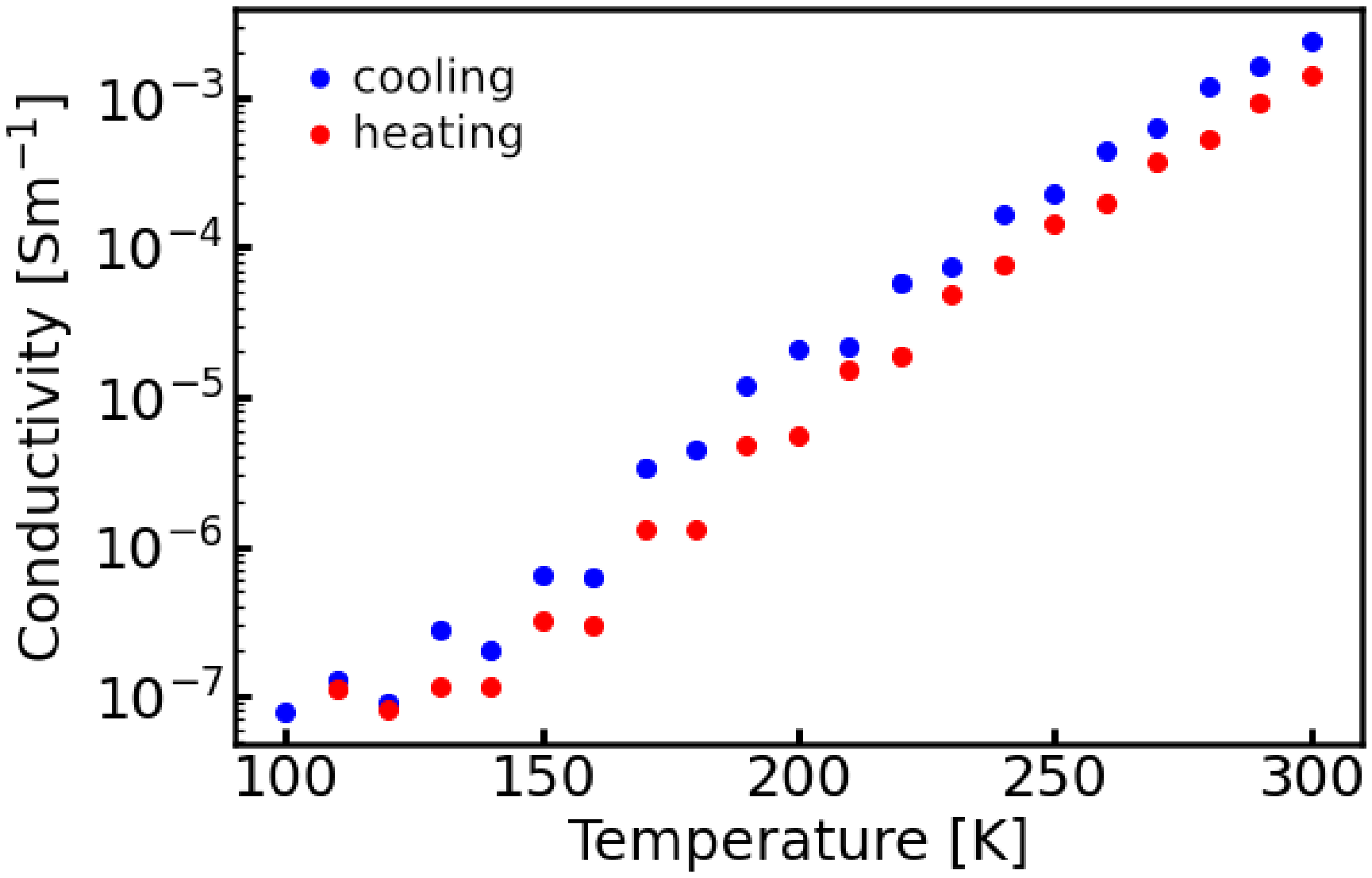

Supplementary Figure 6: Electrical conductivity of Nb_3Cl_8 versus set temperature in a range of 100 K to 300 K. red: cooling; blue: heating

Magnetic measurements

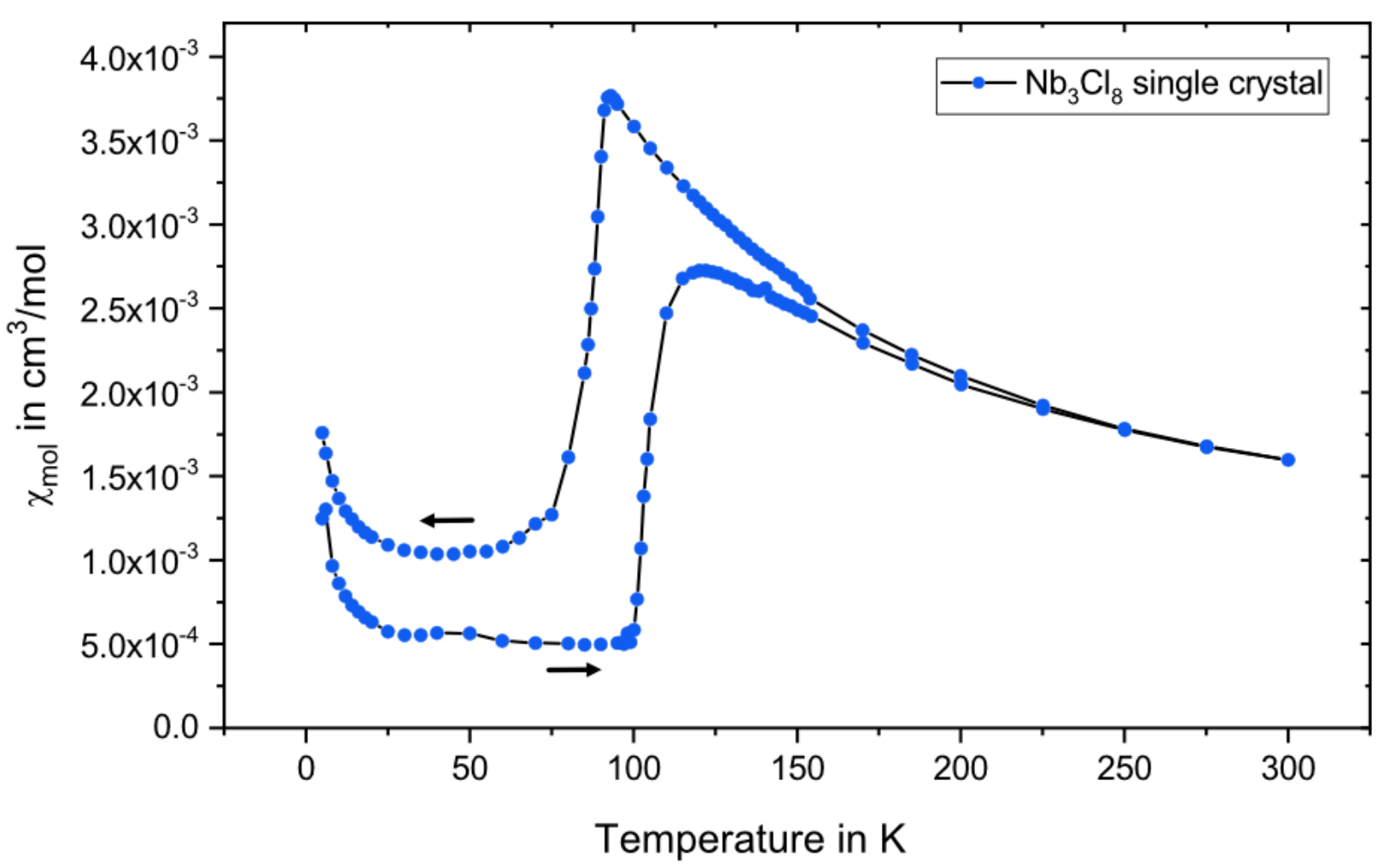

Supplementary Figure 7: Magnetic susceptibility of single crystals of Nb_3Cl_8 versus set temperature in a range of 5 K to 300 K.

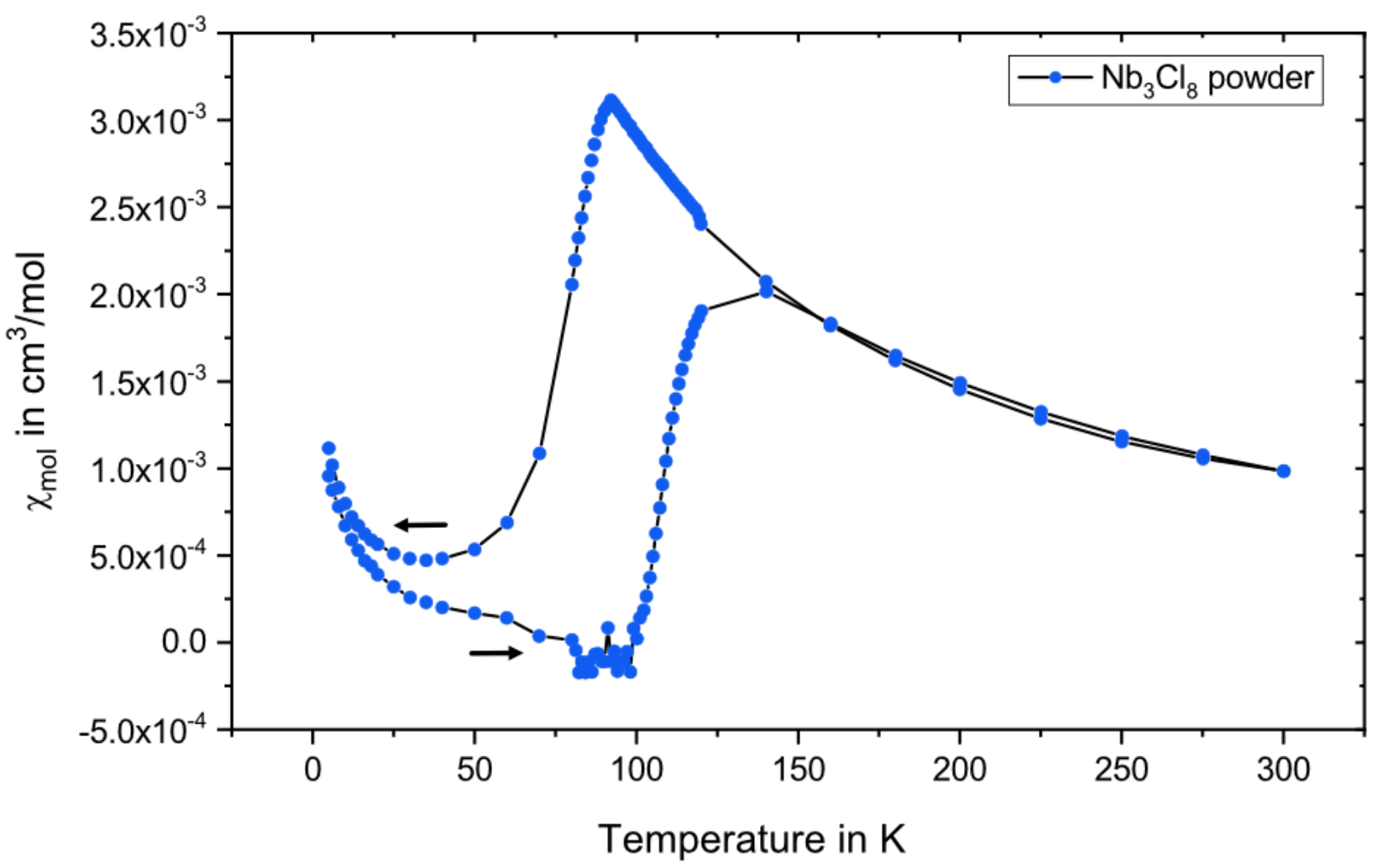

Supplementary Figure 8: Magnetic susceptibility of a powdered sample of Nb_3Cl_8 versus set temperature in a range of 5 K to 300 K.

References

(1) Aryasetiawan, F.; Imada, M.; Georges, A.; Kotliar, G.; Biermann, S.; Lichtenstein, A. I. Frequency-dependent local interactions and low-energy effective models from electronic structure calculations. *Phys. Rev. B* **2004**, *70*, 195104.

(2) Miyake, T.; Aryasetiawan, F. Screened Coulomb interaction in the maximally localized Wannier basis. *Phys. Rev. B* **2008**, *77*, 085122.

(3) Grytsiuk, S.; Katsnelson, M. I.; van Loon, E. G. C. P.; Rösner, M. Nb_3Cl_8: a prototypical layered Mott-Hubbard insulator. *npj Quantum Mater.* **2024**, *9*, 1–13.

(4) Aretz, J.; Grytsiuk, S.; Liu, X.; Feraco, G.; Knekna, C.; Waseem, M.; Dan, Z.; Bianchi, M.; Hofmann, P.; Ali, M. N. et al. From Strong to Weak Correlations in Breathing-Mode Kagome van der Waals Materials: $Nb_3(F,Cl,Br,I)_8$ as a Robust and Versatile Platform for Many-Body Engineering. *Phys. Rev. X* **2025**, *15*, 041042.

(5) Haraguchi, Y.; Michioka, C.; Ishikawa, M.; Nakano, Y.; Yamochi, H.; Ueda, H.; Yoshimura, K. Magnetic–nonmagnetic phase transition with interlayer charge disproportionation of Nb_3 trimers in the cluster compound Nb_3Cl_8. *Inorg. Chem.* **2017**, *56*, 3483–3488.

(6) Sheckelton, J. P.; Plumb, K. W.; Trump, B. A.; Broholm, C. L.; McQueen, T. M. Rearrangement of van der Waals stacking and formation of a singlet state at T = 90 K in a cluster magnet. *Inorg. Chem. Front.* **2017**, *4*, 481–490.

(7) Wendlandt, W. W.; Hecht, H. G. *Reflectance Spectroscopy*; Interscience Publishers, 1966.

(8) Kubelka, P.; Munk, F. An article on optics of paint layers. *Z. Tech. Phys* **1931**, *12*, 259–274.

(9) Kubelka, P. New contributions to the optics of intensely light-scattering materials. Part I. *J. Opt. Soc. Am.* **1948**, *38*, 448–457.

(10) Zanatta, A. R. Revisiting the optical bandgap of semiconductors and the proposal of a unified methodology to its determination. *Sci. Rep.* **2019**, *9*, 11225.
